# Immobilized artificial membrane-chromatographic and computational descriptors in studies of soil-water partition of environmentally relevant compounds

**DOI:** 10.1007/s11356-022-22514-x

**Published:** 2022-08-22

**Authors:** Anna W. Sobańska

**Affiliations:** Department of Analytical Chemistry, Medical University of Łódź, ul. Muszyńskiego 1, 90-151 Lodz, Poland

**Keywords:** Soil-water partition, IAM chromatography, Calculated descriptors, Artificial neural networks, Multiple linear regression

## Abstract

Chromatographic retention factor log *k*_*IAM*_ obtained from immobilized artificial membrane (IAM) HPLC with buffered, aqueous mobile phases and calculated molecular descriptors (molecular weight — log *M*_*W*_; molar volume — *V*_*M*_; polar surface area — *PSA*; total count of nitrogen and oxygen atoms -(*N + O*); count of freely rotable bonds — *FRB*; H-bond donor count — *HD*; H-bond acceptor count — *HA*; energy of the highest occupied molecular orbital — *E*_*HOMO*_; energy of the lowest unoccupied orbital — *E*_*LUMO*_; dipole moment — *DM*; polarizability — *α*) obtained for a group of 175 structurally unrelated compounds were tested in order to generate useful models of solutes’ soil-water partition coefficient normalized to organic carbon log *K*_*oc*_. It was established that log *k*_*IAM*_ obtained in the conditions described in this study is not sufficient as a sole predictor of the soil-water partition coefficient. Simple, potentially useful models based on log *k*_*IAM*_ and a selection of readily available, calculated descriptors and accounting for over 88% of total variability were generated using multiple linear regression (MLR) and artificial neural networks (ANN). The models proposed in the study were tested on a group of 50 compounds with known experimental log *K*_*oc*_ values by plotting the calculated vs. experimental values. There is a good close similarity between the calculated and experimental data for both MLR and ANN models for compounds from different chemical families (*R*^2^ ≥ 0.80, *n* = 50) which proves the models’ reliability.

**Supplementary Information:**

The online version contains supplementary material available at 10.1007/s11356-022-22514-x.

## Introduction

The fate and transport of solutes in the environment depend on their physico-chemical properties such as lipophilicity, volatility, water solubility, and ability to partition between soil and water. The soil-water partition coefficient *K*_*oc*_ (normalized to the soil organic carbon content to reduce the differences among soils) is a very important parameter governing the fate of compounds in the soil-water compartment (Doucette 2003). It influences the chemicals’ mobility in soil, their environmental persistence, and the processes of their removal in wastewater management facilities (Andrić et al. 2016). A very high value of *K*_*oc*_ means a compound is strongly retained by soil and organic matter and does not move throughout the soil. A very low value means a molecule is highly mobile in soil. *K*_*oc*_ is a very important input parameter for estimating environmental distribution and environmental risk related to a chemical substance. A highly mobile substance is more likely to move to groundwater or surface water from soil.

Direct determination of *K*_*oc*_ is based on studies of partitioning phenomena in biphasic soil-water systems using batch or continuous flow experiments. Such tests are, however, tedious and time-consuming, and the results tend to be inconsistent due to experimental difficulties — incomplete separation of phases and volatilization and biological or chemical degradation of solutes (Hodson and Williams 1988). The need to obtain *K*_*oc*_ values very rapidly and for large groups of compounds has led to the development of other methods. Entirely computational approaches are based on molecular descriptors such as water solubility *WS* (Gawlik et al. 1997), octanol-water partition coefficient *P*_*ow*_ (Karickhoff et al. 1979; Hodson and Williams 1988; Pussemier et al. 1990; Müller and Kördel 1996; Gawlik et al. 1997; Doucette 2003), molecular connectivity indices *MCI* (Gawlik et al. 1997; Tao et al. 2001), topological indices (Meylan et al. 1992; Gawlik et al. 1997), or solvation parameters (Gawlik et al. 1997; Poole and Poole 1999; Nguyen et al. 2005; Poole et al. 2013). Alternatively, it is possible to predict soil-water partition using chromatographic descriptors obtained by liquid chromatography on octadecyl-, cyano-, diol-, ethyl-, trimethylammonium-, or aminopropyl-modified silica or on sorbents containing immobilized humic acid or soil (Helling and Turner 1968; Praveen-Kumar et al. 1987; Vowles and Mantoura 1987; Jamet and Thoisy-Dur 1988; Pussemier et al. 1990; Szabo et al. 1990a, b; Kördel et al. 1993, 1995a, b, 1997; Christianson and Howard 1994; Müller and Kördel 1996; Gawlik et al. 1997, 1998, 2000; Poole and Poole 1999; Szabó et al. 1999; Xu et al. 1999, 2002; Ravanel et al. 1999; Guo et al. 2004; Bermúdez-Saldaña et al. 2006; Mrozik et al. 2008; Andrić et al. 2010; Bi et al. 2010; Hidalgo-Rodríguez et al. 2012; Poole et al. 2013; Sobańska 2021). Chromatographic methods of *K*_*oc*_ determination are fast and relatively cheap, with the benefit of high reproducibility, especially when commercially available stationary phases are used. It is generally accepted that log *K*_*oc*_ is connected with the chromatographic retention factor log *k* via a Collander-type linear relationship: log *K*_*oc*_ = *a* + *b* log *k* (Bermúdez-Saldaña et al. 2006). Linear relationships have also been discovered between log *K*_*oc*_ and thin-layer chromatographic descriptors: *R*_*M*_ value defined by Bate-Smith and Westall: *R*_*M*_ = log (1/*R*_*f*_ − 1) (Bate-Smith and Westall 1950) and measured for a single composition of a mobile phase; *R*_*M*_^0^ value obtained from a series of chromatographic experiments with mobile phases containing different concentrations *φ* of a water-miscible solvent (organic modifier) by extrapolation of *R*_*M*_ vs. *φ* plots to zero concentration of the modifier (most frequently by using the linear Soczewiński-Wachmeister equation: *R*_*M*_ = *R*_*M*_^0^ + *S φ* (Soczewiński and Wachtmeister 1962)); the slope *S* taken from the Soczewiński-Wachmeister equation; the real or hypothetical concentration of the organic modifier furnishing *R*_*M*_ = 0 (i.e., *R*_*f*_ = 0.5): *φ*_0_ = −*R*_*M*_^0^/*S*; *PC*_1_ (the 1st principal component computed for thin layer chromatographic retention data). Chromatographic retention parameters are usually considered to be sufficiently good as sole predictors of log *K*_*oc*_, but some authors suggest that other descriptors (e.g., polar surface area, *PSA*) should be incorporated to obtain better fitting models (Sobańska 2021).

Biomimetic liquid chromatography on immobilized artificial membrane (IAM) stationary phases has been used to predict the physicochemical properties and bioavailability of compounds for several years (Sobanska and Brzezinska 2017). IAM chromatographic phases are excellent tools to model drug distribution because of their ability to mimic natural membranes, and the main applications of IAM chromatography are in drug discovery (Valko 2019). The more recent applications of IAM chromatography extend from medicinal to environmental chemistry. Studies of compounds’ bioconcentration in aquatic organisms (Tsopelas et al. 2017, 2018), ecotoxicity of pesticides (Stergiopoulos et al. 2019), and soil-water sorption of herbicides (Hidalgo-Rodríguez et al. 2012) prove that IAM chromatography is a source of valuable information in many areas of research. The objective of the present study was to propose useful models of the soil-water partition coefficients normalized to organic carbon (*K*_*oc*_) using a large group of structurally diverse organic compounds, based their IAM chromatographic and computational descriptors. The *K*_*oc*_ models considered in this study should be applicable during early steps of a drug discovery process, when drugs’ physico-chemical and biological properties are often studied in vitro using IAM chromatography. At this stage, high throughput is more important than accuracy and the chromatographic and computational data are collected for other purposes. It was anticipated that IAM chromatographic and computational studies of soil-water partition of compounds could be carried out concurrently with pharmacokinetic studies.

## Experimental

### Calculated molecular descriptors

The molecular descriptors for compounds investigated during this study were calculated with HyperChem 8.0, utilizing PM3 semi-empirical method with Polak-Ribiere conjugate gradient algorithm for semi-empirical structure optimization (Polak and Ribiere 1969): total dipole moment — *DM* (D), molecular weight — *M*_*w*_ (g mol^−1^), energy of the highest occupied molecular orbital — *E*_*HOMO*_ (eV), and energy of the lowest unoccupied molecular orbital − *E*_*LUMO*_ (eV). Other physicochemical parameters (octanol-water partition coefficient — log *K*_*ow*_, polar surface area — *PSA* (Å^2^), H-bond donor count — *HD*, H-bond acceptor count — *HA*, polarizability — *α* (Å^3^), molar volume — *V*_*M*_ (cm^3^), freely rotable bond count — *FRB*) were calculated using ACD/Labs 8.0 software, using the SMILES codes of molecules as the input data. (*N + O*) — total nitrogen and oxygen atom count was calculated from the molecular structures. The Abraham’s solvation parameters (*A* — hydrogen bond acidity; *B* — hydrogen bond basicity; *S* — dipolar interactions; *E* — excess molar refractivity; *V* — McGowan’s molecular volume) were taken from the paper by Sprunger et al. (2007). The calculated molecular descriptors are given in Table 1 (Supplementary Materials).

### IAM chromatography

The chromatographic retention factors log *k*_*IAM*_ used in this study were compiled by Sprunger at al. (Sprunger et al. 2007). They were obtained on a IAM.PC.DD2 HPLC column using an aqueous mobile phase buffered at pH ≤ 3 for carboxylic acids (to suppress their ionization) and in the pH range 6.5 to 7.5 for other compounds.

### MLR and ANN models

Multiple linear regression (MLR) models were generated using Statistica v. 13, stepwise forward regression mode (*F* to enter set at 1 and *F* to remove set at 0).

Multilayer perceptron (MLP) networks, with the number of inputs the same as the number of variables, the varying number of hidden units, and one output unit, were generated using Statistica v. 13 (regression mode, Automated Network Search — ANS module, 1000 networks to train, 5 networks to retain). The neuron activation functions were selected from the following group: identity, logistic, hyperbolic tangent, and exponential. BFGS (Broyden-Fletcher-Goldfarb-Shanno algorithm) was used to train the network together with the sum of squares (SOS) error function.

## Results and discussion

### Calculated log K_oc_ coefficients

A soil-water partition coefficient normalized to the organic carbon content (*K*_*oc*_) is an important parameter influencing the fate of solutes in the soil-water compartment. In this study, because of the lack of experimental data for the whole studied group of compounds, *K*_*oc*_ was initially calculated using the four most commonly accepted computational approaches (Andrić et al. 2016) (Table 2, Supplementary Materials):

1 $$\log\ {K_{oc}}^{(1)}=0.52\ MCI+0.60+\mathrm{corrections}$$

2 $$\log\ {K_{oc}}^{(2)}=0.55\ \log\ {K}_{ow}+0.93+\mathrm{corrections}$$

3 $$\log\ {K_{oc}}^{(3)}=0.14+0.15\ A-1.98\ B-0.72\ S+1.10\ E+2.28\ V$$

4 $$\log\ {K_{oc}}^{(4)}=0.19-0.23\ A-2.33\ B+0.72\ E+2.12\ V$$

where *MCI* is the first-order molecular connectivity index; log *K*_*ow*_ is the estimated octanol-water partition coefficient (KOWWIN); and *A*, *B*, *S*, *E*, and *V* are the Abraham’s solvation parameters (*A* — hydrogen bond acidity; *B* — hydrogen bond basicity; *S* — dipolar interactions; *E* — excess molar refractivity; *V* — McGowan’s molecular volume). Log *K*_*oc*_^(1)^ and log *K*_*oc*_^(2)^ values were calculated using the EpiSuite software (EpiWeb 4.1, KOCWIN module) (US EPA O 2015).

At this point, attention turned to log *K*_*oc*_^(4)^ as a reference soil-water partition coefficient value readily available for the whole group of studied solutes. The known theoretical log *K*_*oc*_ models (1) to (4) have their limitations: e.g., linear models based on log *K*_*ow*_ (log *K*_*oc*_^(2)^) are not particularly useful for polar or ionizable solutes (Franco and Trapp 2008). However, Eq. (4) appears to be applicable to compounds across a relatively wide range of chemical families, including ionizable or polar molecules (e.g., ionizable or polar compounds within the studied group, whose experimental log *K*_*oc*_ values are available (US EPA O 2015) — ethanol, *35*; acetic acid, *98*; phenylacetic acid, *122* and benzoic acid, *144*).

### Calculated log K_oc_ coefficients vs. IAM chromatographic descriptors

The values of log *K*_*oc*_ calculated according to Eq. (4) were plotted against the IAM retention factor log *k*_*IAM*_. The linear relationship between log *K*_*oc*_^(4)^ and log *k*_*IAM*_ (Eq. (5a)) accounts for 81% of total log *K*_*oc*_ variability.

5a $${\displaystyle \begin{array}{l}\log\ {K_{oc}}^{(4)}=1.04\ \left(\pm 0.05\right)+0.84\ \left(\pm 0.03\right)\log\ {k}_{IAM}\\ {}\left(n=175,\kern0.5em {R}^2=0.81,\kern0.75em {R^2}_{\mathrm{adj}}=0.81,F=729.3,p<0.01,{s}_e=0.42,\mathrm{aqueous}\ \mathrm{mobile}\ \mathrm{phase}\right)\end{array}}$$

The results of log *K*_*oc*_ modeling using a single chromatographic descriptor (log *k*_*IAM*_) obtained in this study (Eq. (5a)) are slightly poorer than those reported by Hidalgo-Rodríguez et al. (2012), who found the IAM retention factor superior to other chromatographic measures of soil-water partition. However, Hidalgo-Rodrigues et al. generated their IAM-chromatographic model (Eq. (5b)) using a significantly smaller group of compounds (*n* = 38) of much more limited structural diversity:

5b $${\displaystyle \begin{array}{l}\log\ {K}_{oc}=2.20\ \left(\pm 0.05\right)+2.17\left(\pm 0.12\right)\ \log\ {k}_{IAM}\\ {}\left(n=38,\kern0.5em {R}^2=0.89,F=303,\kern0.5em \mathrm{mobile}\ \mathrm{phase}:\mathrm{acetonitrile}\ 40\%\left(\mathrm{v}/\mathrm{v}\right)\right)\end{array}}$$

The group of compounds considered in the current study contains solutes of very different physico-chemical properties, e.g., molecules that are relatively strong organic acids and bases, from different chemical families and of different usage. The studied group contains simple organic chemicals (e.g., solvents), drugs (including, e.g., steroids, non-steroid anti-inflammatory drugs, topical anesthetics, beta-blockers, antibacterial and antiparasitic drugs), and components of fragrances/essential oils. Some of the studied compounds are likely to be released to the environment (e.g., by washing off the skin or with feces) and seriously affect the wildlife (e.g., by interacting with the reproductive processes of aquatic animals).

### Multiple linear regression studies

In the current study, compared to the previous investigations (Hidalgo-Rodríguez et al. 2012), the focus is on the possibility of making the chromatographic method of *K*_*oc*_ prediction using the IAM retention factor more universal by incorporating additional, easily calculated molecular descriptors, and by considering both linear and non-linear relationships between the independent variables and the studied property, rather than on selecting the most effective chromatographic system.

**Fig. 1:**
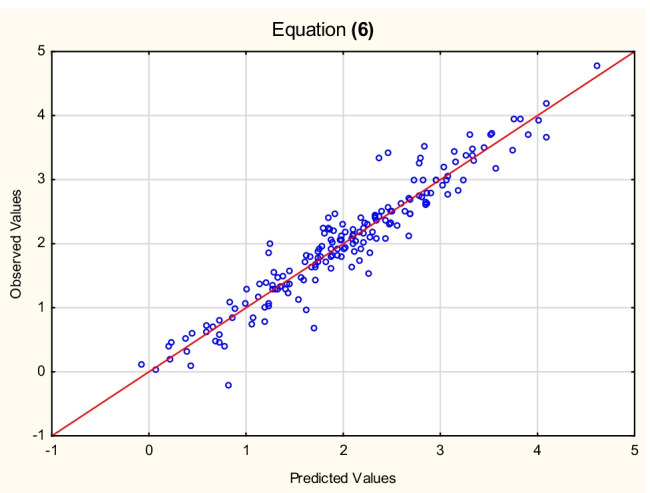
Equation (6) — log *K*_*oc*_^(4)^ vs. log *K*_*oc*_^(6)^ values (Table 3, Supplementary Materials)

Equation (5a) accounts for only 81% of total log *K*_*oc*_ variability; attempts were therefore made to improve a model by incorporating additional independent variables. However, this was not an easy task, since log *k*_*IAM*_ already encodes some properties, responsible for partition and transport phenomena (especially lipophilicity expressed as log *K*_ow_ — the correlation between log *K*_*ow*_ and log *k*_*IAM*_ is linear, with *R*^2^ = 0.84). When lipophilicity and redundant variables defining the molecular size were excluded from the analysis, Eq. (6) containing the following independent variables: log *k*_*IAM*_, log *M*_*w*_, (*N + O*), *E*_*LUMO*_, *α*, *E*_*HOMO*_, *HA*, *PSA*, *HD*, and *DM* was generated by forward stepwise multiple regression (Fig. 1):

6 $${\displaystyle \begin{array}{l}\log\ {K_{oc}}^{(4)}=-0.80\ \left(\pm 0.74\right)+0.54\ \left(\pm 0.04\right)\ \log\ {k}_{IAM}+0.86\ \left(\pm 0.37\right)\ \log\ {M}_w-0.016\ \left(\pm 0.058\right)\ \left(N+O\right)-0.12\ \left(\pm 0.04\right)\ {E}_{LUMO}\ +0.032\ \left(\pm 0.008\right)\ \alpha -0.0015\ \left(\pm 0.0365\right)\ {E}_{HOMO}-0.21\ \left(\pm 0.08\right)\ HA+0.0098\ \left(\pm 0.0036\right)\ PSA\ -0.12\ \left(\pm 0.05\right)\ HD+0.019\ \left(\pm 0.017\right)\ DM\ \\ {}\left(n=175,{R}^2=0.91,{R^2}_{\mathrm{adj}}=0.91,F=171.8,p<0.01,{s}_e=0.29\right)\end{array}}$$

The group of 175 studied compounds was divided into two subsets: a training set (*n* = 125) and a test set (*n* = 50, compounds with known log *K*_*oc*_^exp^ values). Equation (7), generated for the training set, and containing the same variables as Eq. (6), is as follows:

7 $${\displaystyle \begin{array}{l}\log\ {K_{oc}}^{(4)}=-0.25\ \left(\pm 1.02\right)+0.54\ \left(\pm 0.04\right)\ \log\ {k}_{IAM}+0.72\ \left(\pm 0.48\right)\ \log\ {M}_w-0.0044\ \left(\pm 0.0624\right)\ \left(N+O\right)-0.13\ \left(\pm 0.05\right)\ {E}_{LUMO}+0.036\ \left(\pm 0.010\right)\ \alpha +0.032\ \left(\pm 0.046\right)\ {E}_{HOMO}-0.33\ \left(\pm 0.09\right)\ HA+0.017\ \left(\pm 0.004\right)\ PSA-0.17\ \left(\pm 0.06\right)\ HD+0.016\ \left(\pm 0.021\right)\ DM\ \\ {}\left(n=125,{R}^2=0.92,{R^2}_{\mathrm{adj}}=0.91,F=124.9,p<0.01,{s}_e=0.30\right)\end{array}}$$

The values of log *K*_*oc*_^(7)^ were calculated for the test set according to Eq. (7) and plotted against the reference log *K*_*oc*_^(4)^ values to furnish a linear relationship (*R*^2^ = 0.86, or *R*^2^ = 0.93 when thiourea was removed as an outlier). The model (6) was also tested on the same test set of 50 compounds, whose log *K*_*oc*_^exp^ values were available (US EPA O 2015). The resulting relationship between log *K*_*oc*_^(6)^ and log *K*_*oc*_^exp^ is linear, with *R*^2^ = 0.85.

**Fig. 2:**
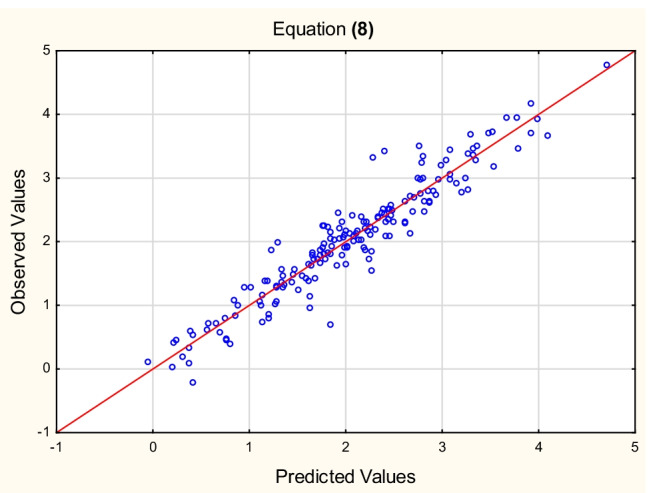
Equation (8) — log *K*_*oc*_^(4)^ vs. log *K*_*oc*_^(8)^ values (Table 3, Supplementary Materials)

**Fig. 3:**
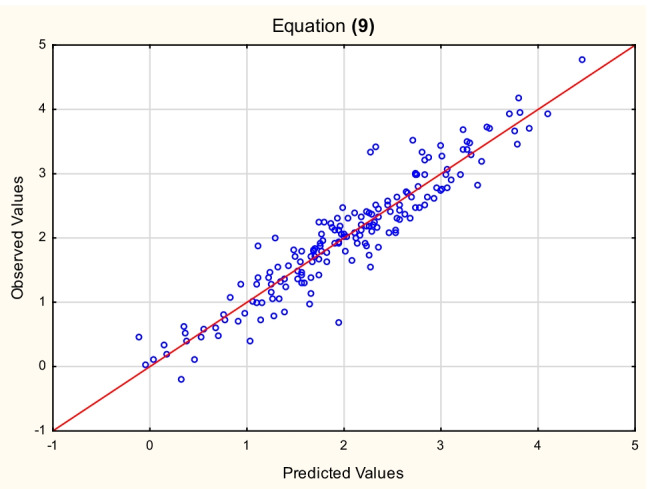
Equation (9) — log *K*_*oc*_^(4)^ vs. log *K*_*oc*_^(9)^ values (Table 3, Supplementary Materials)

**Fig. 4:**
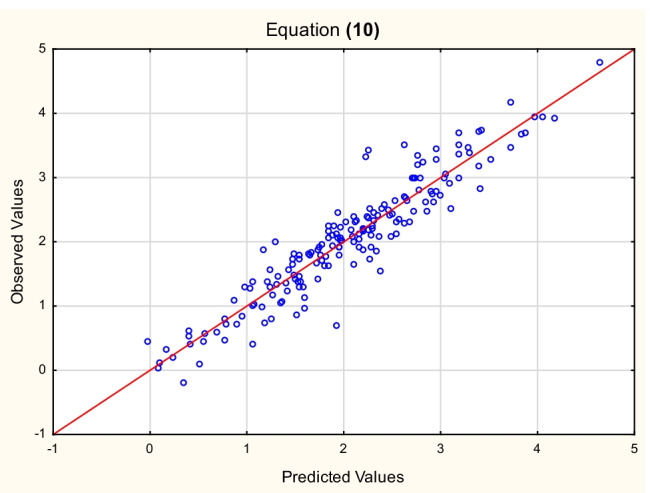
Equation (10) — log *K*_*oc*_^(4)^ vs. log *K*_*oc*_^(10)^ values (Table 3, Supplementary Materials)

Equation (6), despite encouraging results of validation, was found unsatisfying because it seems unpractical and over-parameterized — it contains 10 independent variables whose contributions (apart from log *k*_*IAM*_ and log *M*_*w*_) are not very significant. At this point, it was decided to simplify Eq. (6) as much as possible; the attempts to achieve it furnished Eqs. (8), (9), and (10), based on reduced sets of independent variables (Figs. 2, 3 and 4).

8 $${\displaystyle \begin{array}{l}\log\ {K_{oc}}^{(4)}=-0.96\ \left(\pm 0.74\right)+0.58\ \left(\pm 0.03\right)\ \log\ {k}_{IAM}+0.66\ \left(\pm 0.37\right)\ \log\ {M}_w-0.090\ \left(\pm 0.021\right)\ \left(N+O\right)-0.16\ \left(\pm 0.03\right)\ {E}_{LUMO}+ 0.030\ \left(\pm 0.008\right)\ \alpha -0.058\ \left(\pm 0.028\right)\ {E}_{HOMO}\ \\ {}\left(n=175,{R}^2=0.91,{R^2}_{\mathrm{adj}}=0.90,F=270.0,p<0.01,{s}_e=0.30\right)\end{array}}\kern1em$$

9 $${\displaystyle \begin{array}{l}\log\ {K_{oc}}^{(4)}=-2.85\ \left(\pm 0.40\right)+0.60\ \left(\pm 0.03\right)\ \log\ {k}_{IAM}+1.99\ \left(\pm 0.21\right)\ \log\ {M}_w-0.056\ \left(\pm 0.020\right)\ \left(N+O\right)\ \\ {}\left(n=175,{R}^2=0.89,{R^2}_{\mathrm{adj}}=0.89,F=460.9,p<0.01,{s}_e=0.32\right)\end{array}}\kern1em$$

10 $${\displaystyle \begin{array}{l}\log\ {K_{oc}}^{(4)}=-2.06\ \left(\pm 0.29\right)+0.65\ \left(\pm 0.03\right)\ \log\ {k}_{IAM}+1.54\ \left(\pm 0.14\right)\ \log\ {M}_w\ \\ {}\left(n=175,{R}^2=0.88,{R^2}_{\mathrm{adj}}=0.88,F=661.0,p<0.01,{s}_e=0.32\right)\end{array}}\kern1em$$

The group of 175 studied compounds was divided into two subsets: a training set (*n* = 125) and a test set (*n* = 50, compounds with known log *K*_*oc*_^exp^ values). Equations (11), (12), and (13) generated for the training set, and containing the same variables as Eqs. (8), (9), and (10) are as follows:

11 $${\displaystyle \begin{array}{l}\log\ {K_{oc}}^{(4)}=-1.33\ \left(\pm 1.04\right)+0.57\ \left(\pm 0.04\right)\log\ {k}_{IAM}+0.77\ \left(\pm 0.50\right)\log\ {M}_w-0.10\ \left(\pm 0.03\right)\left(N+O\right)-0.18\ \left(\pm 0.04\right){E}_{LUMO}+0.029\ \left(\pm 0.011\right)\ \alpha -0.076\ \left(\pm 0.037\right)\ {E}_{HOMO}\ \\ {}\left(n=125,{R}^2=0.90,{R^2}_{\mathrm{adj}}=0.90,F=181.7,p<0.01,{s}_e=0.32\right)\end{array}}\kern1em$$

12 $${\displaystyle \begin{array}{l}\log\ {K_{oc}}^{(4)}=-3.13\ \left(\pm 0.49\right)+0.58\ \left(\pm 0.04\right)\log\ {k}_{IAM}+2.14\ \left(\pm 0.26\right)\ \log\ {M}_w-0.067\left(\pm 0.024\right)\left(N+O\right)\\ {}\left(n=125,{R}^2=0.88,{R^2}_{\mathrm{adj}}=0.88,F=306.4,p<0.01,{s}_e=0.35\right)\end{array}}\kern1em$$

13 $${\displaystyle \begin{array}{l}\log\ {K_{oc}}^{(4)}=-2.22\ \left(\pm 0.36\right)+0.64\ \left(\pm 0.04\right)\ \log\ {k}_{IAM}+1.62\ \left(\pm 0.18\right)\ \log\ {M}_w\\ {}\left(n=125,{R}^2=0.88,{R^2}_{\mathrm{adj}}=0.87,F=432.8,p<0.01,{s}_e=0.36\right)\end{array}}\kern1em$$

The relationships between the log *K*_*oc*_ values calculated for the test set according to Eqs. (11), (12), and (13) and the reference values — log *K*_*oc*_^(4)^ are linear (*R*^2^ = 0.90, 0.89, and 0.90, respectively). The models (8), (9), and (10) were also tested on the same test set of 50 compounds, whose log *K*_*oc*_^exp^ values were available (US EPA O 2015). The resulting relationships between the predicted and experimental log *K*_*oc*_ values are linear, with *R*^2^ = 0.83, 0.80, and 0.80, respectively.

Equation (9) contains, apart from log *k*_*IAM*_ and log *M*_*W*_, the total N and O atom count (*N + O*). This descriptor is a relatively good measure of polarity and H-bonding activity of molecules, and it has been used to predict their ADME properties, e.g., the blood-brain barrier permeability (Clark 2003) or skin permeability (Sobańska et al. 2021). However, it plays a rather minor role in soil-water partition modeling using MLR and Eq. (10) seems to be sufficient for rapid log *K*_*oc*_ predictions.

### Artificial neural network analysis

At this point, attention turned to artificial neural network (ANN) models. Artificial neural networks are widely used to predict drugs’ bioavailability (Carracedo-Reboredo et al. 2021) or properties such as an affinity for phospholipids using IAM chromatography and calculated descriptors (Ciura et al. 2021). The great advantages of neural networks compared to MLR are the possibility of utilizing both linear and non-linear relationships between input data and a predicted parameter and the ability of ANNs to learn these relationships directly from the data being modeled.

In this study, the ANN models were built for the same group of compounds (*n* = 175) that were used in the MLR analysis. 125 substances without the known log *K*_*oc*_^exp^ data were used as a training set; the remaining compounds, whose log *K*_*oc*_^exp^ were available (US EPA O 2015) were divided into a test set (*n* = 25) and a validation set (*n*= 25).

ANNs make it possible to process a large number of descriptors that can be easily obtained using readily available software. The selection of ANN input data is an important step because if the number of parameters is excessive considering the number of cases, overfitting may occur. An over-fitted model fits perfectly the data used as a training set but is likely to be inapplicable to additional cases (a simple example of an over-fitted model is a polynomial of a degree equal or close to the number of data points which fits the existing data very well but is not very good at generalization while extrapolated beyond the fitted data). In this study, a decision was made to consider, apart from the IAM retention factor log *k*_*IAM*_, a relatively small number of parameters, especially those that are known to be good predictors of environmental phenomena (Mamy et al. 2015). According to Mammy et al., 686 different molecular descriptors are found in 790 QSAR equations generated to predict 90 environmental parameters, but the descriptors that are most widely used are *E*_*HOMO*_, *E*_*LUMO*_, *M*_*w*_, *α*, and *DM*. The input data used in this part of the study were selected from the following group, involved earlier in multiple regression analysis: log *k*_*IAM*_, log *M*_*w*_, *PSA*, (*N + O*), *HD*, *HA*, *α*, *DM*, *E*_*HOMO*_, and *E*_*LUMO*_, and the predicted variable was log *K*_*oc*_^(4)^. In the case of every ANN model, 1000 networks were generated and five with the smallest error were retained for further examination in search for a network that would give the results being in the best agreement with the reference data (log *K*_*oc*_^(4)^) for the whole group of studied compounds (*n* = 175) and with the experimental data (log *K*_*oc*_^exp^) for a subgroup of cases (*n* = 50). The ANN models investigated at this stage of the study involved the following sets of independent variables, used earlier in the multiple regression analysis (Eqs. (6), (8), (9), and (10)) (Table 4, Supplementary Materials):

- ANN_1_: log *k*_*IAM*_, log *M*_*w*_, *PSA*, (*N + O*), *HD*, *HA*, *α*, *DM*, *E*_*HOMO*_, *E*_*LUMO*_
- ANN_2_: log *k*_*IAM*_, log *M*_*w*_, (*N + O*), *α*, *E*_*HOMO*_, *E*_*LUMO*_
- ANN_3_: log *k*_*IAM*_, log *M*_*w*_, (*N + O*)
- ANN_4_: log *k*_*IAM*_, log *M*_*w*_

The details of all the ANNs considered at this stage of the study are given in Table 4 (Supplementary Materials). The networks ANN_1_ to ANN_4_ were studied using a tool known as global sensitivity analysis (GSA), which rates the importance of the models’ input variable by computing sums of squared residuals for the model when the respective predictor is eliminated, compared to the full model. When an input variable scores 1 or less in GSA, it means that this particular network would perform better if this variable is omitted; however, no such situation occurred for the networks ANN_1_ to ANN_4_. All the 20 most promising networks were analyzed by plotting the predicted log *K*_*oc*_^ANN^ values against the reference log *K*_*oc*_^(4)^ values obtained for the whole group of 175 studied compounds. The predicted log *K*_*oc*_^ANN^ values were also plotted against the experimental log *K*_*oc*_^exp^ values for 50 compounds, whose log *K*_*oc*_^exp^ values are available. The resulting dependences between log *K*_*oc*_^ANN^ and log *K*_*oc*_^(4)^ and between log *K*_*oc*_^ANN^ and log *K*_*oc*_^exp^ are linear (see Table 4, Supplementary Materials). It was found that the networks of the ANN_2_ family have the predictive abilities comparable to those of ANN_1_ networks, but since they involve a smaller number of input variables and hidden layers, they are likely to be less prone to overfitting. ANN_4_ networks, based on just two input variables (log *k*_*IAM*_ and log *M*_*w*_), give the prediction results comparable to those obtained using the ANN_3_ networks; similar to MLR models (Eqs. (9) and (10)), it seems that just two variables, log *k*_*IAM*_ and log *M*_*w*_, account for *ca.* 88–89% of the total log *K*_*oc*_ variability (depending on the ANN’s architecture).

**Fig. 5:**
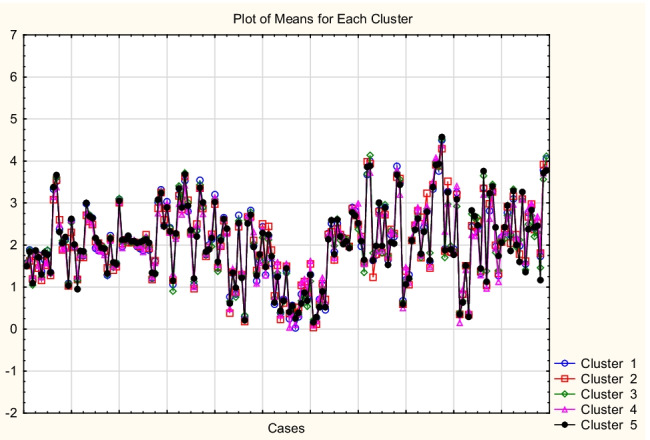
Means for 5 clusters, *n* = 175

**Fig. 6:**
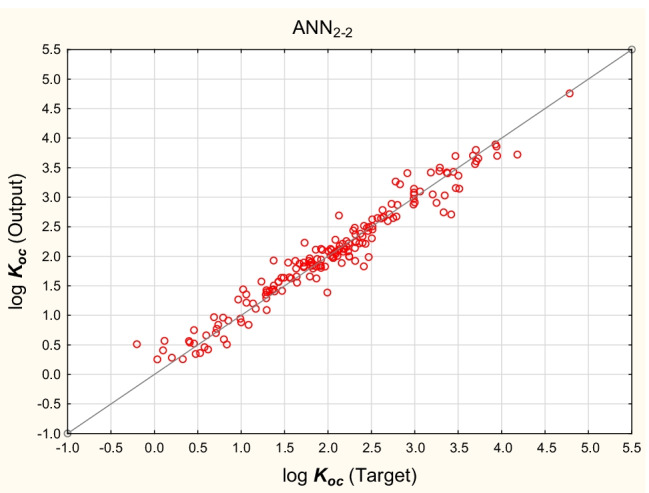
ANN_2-2_ − log *K*_*oc*_^(4)^ vs. log *K*_*oc*_^ANN2-2^ values

The differences and similarities between the reference values (log *K*_*oc*_^(4)^) and the predicted values obtained according to MLR and ANN models described above were studied briefly using Cluster Analysis (*k*-means method). 25 log *K*_*oc*_ values (log *K*_*oc*_^(4)^ and the values predicted using MLR and ANN models: log *K*_*oc*_^(6)^, log *K*_*oc*_^(8)^ to log *K*_*oc*_^(10)^, log *K*_*oc*_^ANN1^, log *K*_*oc*_^ANN2^, log *K*_*oc*_^ANN3^, and log *K*_*oc*_^ANN4^) obtained for 175 compounds were separated between 5 clusters with minimum within-cluster variances and maximum variances between clusters (Table 5, Supplementary Materials). It was established that the reference values (log *K*_*oc*_^(4)^) form a cluster with the values calculated using ANN_2-1_, ANN_2-2_, ANN_2-3_, and ANN_2-4_ networks (Cluster 5). The mean log *K*_*oc*_ values obtained for clusters 1 to 5 were plotted against the experimental log *K*_*oc*_^exp^ values for 50 compounds whose experimental log *K*_*oc*_^exp^ values are available. The resulting relationships are linear (*R*^2^ = 0.84, 0.79, 0.85, 0.80, and 0.84, respectively). The differences between the prediction results for clusters 1 to 5 are relatively small (Fig. 5); for the great majority of studied compounds *1* to *175*, the mean values of log *K*_*oc*_ calculated for all the clusters are similar (S.D. below 0.15, Table 6, Supplementary Materials). However, a network that attracts particular attention is ANN_2-2_ (Fig. 6) based on 6 input variables and giving the prediction results superior to those obtained using the MLR model involving the same set of independent variables (Eq. (8)). The values of log *K*_*oc*_^ANN2-2^ calculated using this network are in agreement with the reference values log *K*_*oc*_^(4)^ (*R*^2^ = 0.95, *n* = 175) and with the experimental values log *K*_*oc*_^exp^ (*R*^2^ = 0.86, *n* = 50).

## Conclusions

In this study, new models of the soil-water partition coefficient *K*_*oc*_ were developed using IAM chromatographic retention factors and calculated physico-chemical parameters of 175 structurally diverse compounds. The relationships between log *K*_*oc*_ and the independent variables were either linear (multiple linear regression, MLR models) or non-linear (artificial neural network, ANN models).

Due to the limited availability of experimental permeability data for the solutes investigated in this study, the reference soil-water partition coefficients were calculated according to a widely accepted theoretical model based on Abraham’s solvation parameters. The values of log *K*_*oc*_ obtained using this model are in agreement with the experimental data (where available).

Both MLR and ANN models proposed in this study have certain advantages. Compared to the MLR model, the ANN model gives slightly better prediction results which indicate non-linearity and complexity of correlations between the studied descriptors and the soil-water partition coefficient. The main descriptors, responsible for the variability of log *K*_*oc*_ in MLR equations, are log *k*_*IAM*_ and log *M*_*w*_*.* When other independent variables are incorporated, the ANN models gain more in predictive power than MLR equations. Non-linear modeling is promising in terms of predicting compounds’ mobility in soil with high accuracy. However, linear relationships (Eq. (10)) give sufficiently good results and have the benefit of simplicity. Linear models are also more intuitive and can be easily applied during early steps of a drug discovery process. At this stage, high throughput is essential; IAM chromatographic and computational descriptors are collected to study drugs’ pharmacokinetic properties, so acquisition of input data for soil mobility studies would not require any additional effort. IAM chromatographic and computational studies of soil-water partition of compounds may therefore be a valuable extension of ADMET studies.

## Supplementary information

ESM 1 (DOCX 72 kb)

ESM 2 (DOCX 28 kb)

ESM 3 (DOCX 99 kb)

ESM 4 (DOCX 19 kb)

ESM 5 (DOCX 12 kb)

ESM 6 (DOCX 32 kb)

## Data Availability

Data used in this manuscript are given as Supplementary Materials or in references cited in the text.

## Declarations

### Studies in humans and animals

No human subjects or animal experiments were involved during the preparation of this manuscript.
