# Supplementary material for: Immobilized artificial membrane-chromatographic and computational descriptors in studies of soil-water partition of environmentally relevant compounds
Source: Environ Sci Pollut Res Int. 2022 Aug 22;30(3):6192–200. doi: 10.1007/s11356-022-22514-x (PMC9895004; doi:10.1007/s11356-022-22514-x)
Supplement: Supplementary file 1 — (DOCX 72 kb) [file 11356_2022_22514_MOESM1_ESM.docx]

Table 1. Calculated physico-chemical properties and log ***k_IAM_*** of compounds ***1*** - ***175***

|  |  | log ***k_IAM_*** | ***M_w_***  [g/ mol] | log ***M_w_*** | ***PSA***  [Å^2^] | log ***K_ow_*** | ***FRB*** | ***N+O*** | ***HD*** | ***HA*** | ***V_M_*** [cm^3^] | ***α***  [Å^3^] | ***DM***  [D] | ***E_HOMO_***  [eV] | ***E_LUMO_***  [eV] |
| --- | --- | --- | --- | --- | --- | --- | --- | --- | --- | --- | --- | --- | --- | --- | --- |
| ***1*** | acetanilide | 0.48 | 135.2 | 2.13 | 29.1 | 1.08 | 1 | 2 | 1 | 2 | 122.5 | 16.07 | 2.89 | -8.82 | -0.03 |
| ***2*** | acetophenone | 0.76 | 120.2 | 2.08 | 17.1 | 1.66 | 1 | 1 | 0 | 1 | 121.0 | 14.38 | 2.80 | -10.00 | -0.44 |
| ***3*** | 2-aminophenol | 0.28 | 109.1 | 2.04 | 46.3 | 0.44 | 2 | 2 | 3 | 2 | 90.1 | 12.83 | 2.34 | -8.10 | 0.52 |
| ***4*** | anisole | 1.06 | 108.1 | 2.03 | 9.2 | 2.13 | 1 | 1 | 0 | 1 | 113.4 | 13.05 | 1.08 | -9.11 | 0.35 |
| ***5*** | benzaldehyde | 0.63 | 106.1 | 2.03 | 17.1 | 1.64 | 1 | 1 | 0 | 1 | 101.1 | 13.08 | 2.69 | -10.05 | -0.48 |
| ***6*** | benzamide | 0.12 | 121.1 | 2.08 | 43.1 | 0.74 | 1 | 2 | 2 | 2 | 108.1 | 13.95 | 3.54 | -9.72 | -0.36 |
| ***7*** | benzene | 0.96 | 78.1 | 1.89 | 0.0 | 2.22 | 0 | 0 | 0 | 0 | 89.4 | 10.41 | 0.00 | -9.75 | 0.40 |
| ***8*** | benzonitrile | 0.77 | 103.1 | 2.01 | 23.8 | 1.65 | 0 | 1 | 0 | 1 | 100.0 | 12.42 | 3.61 | -10.10 | -0.58 |
| ***9*** | benzyl alcohol | 0.35 | 108.1 | 2.03 | 20.2 | 1.03 | 2 | 1 | 1 | 1 | 109.3 | 12.09 | 1.45 | -9.58 | 0.33 |
| ***10*** | biphenyl | 2.88 | 154.2 | 2.19 | 0.0 | 3.98 | 0 | 0 | 0 | 0 | 154.7 | 20.16 | 0.00 | -8.92 | -0.36 |
| ***11*** | 1-bromonaphtalene | 2.90 | 207.1 | 2.32 | 0.0 | 4.22 | 0 | 0 | 0 | 0 | 139.7 | 20.53 | 1.13 | -8.99 | -0.65 |
| ***12*** | 3-bromophenol | 1.71 | 173.0 | 2.24 | 20.2 | 2.63 | 1 | 1 | 1 | 1 | 104.1 | 14.20 | 0.46 | -9.41 | -0.13 |
| ***13*** | cinnamyl alcohol | 1.02 | 134.2 | 2.13 | 20.2 | 1.70 | 3 | 1 | 1 | 1 | 127.9 | 17.32 | 1.47 | -8.95 | -0.15 |
| ***14*** | coumarin | 0.99 | 146.2 | 2.16 | 26.3 | 1.39 | 0 | 2 | 0 | 2 | 117.1 | 15.76 | 4.42 | -9.45 | -0.99 |
| ***15*** | cyclohexanone | 0.08 | 98.2 | 1.99 | 17.1 | 0.76 | 0 | 1 | 0 | 1 | 103.0 | 11.02 | 2.81 | -10.48 | 0.85 |
| ***16*** | diethyl phtalate | 1.63 | 222.1 | 2.35 | 52.6 | 2.70 | 6 | 4 | 0 | 4 | 198.2 | 23.42 | 0.23 | -10.24 | -0.93 |
| ***17*** | 2,6-dimethylphenol | 1.25 | 122.2 | 2.09 | 20.2 | 2.40 | 1 | 1 | 1 | 1 | 120.4 | 14.98 | 1.18 | -8.96 | 0.29 |
| ***18*** | 4-hydroxybenzyl alcohol | 0.12 | 124.1 | 2.09 | 40.5 | 0.30 | 3 | 2 | 2 | 2 | 101.7 | 13.71 | 1.60 | -9.06 | 0.31 |
| ***19*** | 2-methylphenol | 1.02 | 108.1 | 2.03 | 20.2 | 1.94 | 1 | 1 | 1 | 1 | 104.1 | 13.07 | 1.36 | -9.06 | 0.28 |
| ***20*** | 4-methylphenol | 1.02 | 108.1 | 2.03 | 20.2 | 1.94 | 1 | 1 | 1 | 1 | 104.1 | 13.07 | 1.22 | -8.95 | 0.33 |
| ***21*** | naphtalene | 2.47 | 128.2 | 2.11 | 0.0 | 3.45 | 0 | 0 | 0 | 0 | 123.6 | 17.48 | 0.00 | -8.84 | -0.41 |
| ***22*** | 1-naphthol | 2.08 | 144.2 | 2.16 | 20.2 | 2.71 | 1 | 1 | 1 | 1 | 122.0 | 18.23 | 0.90 | -8.54 | -0.36 |
| ***23*** | 2-naphthol | 1.93 | 144.2 | 2.16 | 20.2 | 2.71 | 1 | 1 | 1 | 1 | 122.0 | 18.23 | 0.87 | -8.72 | -0.45 |
| ***24*** | 2-nitroaniline | 1.13 | 138.1 | 2.14 | 71.8 | 1.83 | 2 | 4 | 2 | 4 | 103.6 | 14.68 | 5.24 | -8.75 | -0.82 |
| ***25*** | 4-nitroaniline | 1.01 | 138.1 | 2.14 | 71.8 | 1.39 | 2 | 4 | 2 | 4 | 103.6 | 14.68 | 7.84 | -9.00 | -0.78 |
| ***26*** | nitrobenzene | 1.04 | 123.1 | 2.09 | 45.8 | 1.95 | 1 | 3 | 0 | 3 | 101.3 | 13.00 | 5.25 | -10.60 | -1.14 |
| ***27*** | 4-nitrobenzyl alcohol | 0.72 | 153.1 | 2.19 | 66.1 | 0.76 | 3 | 4 | 1 | 4 | 115.1 | 15.56 | 6.44 | -10.52 | -1.13 |
| ***28*** | 1-nitrobutane | 0.42 | 103.1 | 2.01 | 45.8 | 1.40 | 3 | 3 | 0 | 3 | 107.3 | 10.55 | 4.24 | -12.01 | 0.06 |
| ***29*** | 4-nitrotoluene | 1.35 | 137.1 | 2.14 | 45.8 | 2.41 | 1 | 3 | 0 | 3 | 117.6 | 14.91 | 5.73 | -10.47 | -1.11 |
| ***30*** | phenol | 0.68 | 94.1 | 1.97 | 20.2 | 1.48 | 1 | 1 | 1 | 1 | 87.9 | 11.15 | 1.14 | -9.18 | 0.29 |
| ***31*** | 2-phenylethanol | 0.53 | 122.2 | 2.09 | 20.2 | 1.36 | 3 | 1 | 1 | 1 | 119.8 | 14.80 | 1.39 | -9.55 | 0.29 |
| ***32*** | 4-phenylphenol | 2.56 | 170.2 | 2.23 | 20.2 | 3.20 | 2 | 1 | 1 | 1 | 153.2 | 20.90 | 1.12 | -8.64 | -0.34 |
| ***33*** | toluene | 1.40 | 92.1 | 1.96 | 0.0 | 2.68 | 0 | 0 | 0 | 0 | 105.7 | 12.32 | 0.26 | -9.44 | 0.45 |
| ***34*** | 4-chlorophenol | 1.48 | 128.6 | 2.11 | 20.2 | 2.43 | 1 | 1 | 1 | 1 | 99.8 | 13.09 | 1.39 | -9.01 | 0.05 |
| ***35*** | 2,3-benzofuran | 1.50 | 118.1 | 2.07 | 13.1 | 2.67 | 0 | 1 | 0 | 1 | 106.3 | 14.43 | 0.57 | -9.07 | -0.15 |
| ***36*** | 2,3-dimethylphenol | 1.44 | 122.2 | 2.09 | 20.2 | 2.40 | 1 | 1 | 1 | 1 | 120.4 | 14.98 | 1.62 | -9.00 | 0.29 |
| ***37*** | 2,4-dimethylphenol | 1.46 | 122.2 | 2.09 | 20.2 | 2.40 | 1 | 1 | 1 | 1 | 120.4 | 14.98 | 1.40 | -8.86 | 0.31 |
| ***38*** | 2-nitroanisole | 1.09 | 153.1 | 2.19 | 55.1 | 1.73 | 2 | 4 | 0 | 4 | 125.3 | 15.65 | 5.54 | -10.29 | -0.89 |
| ***39*** | 2-chloroaniline | 1.21 | 127.6 | 2.11 | 26.0 | 1.91 | 1 | 1 | 2 | 1 | 103.7 | 14.03 | 1.53 | -8.17 | 0.31 |
| ***40*** | 3-nitroaniline | 0.93 | 138.1 | 2.14 | 71.8 | 1.37 | 2 | 4 | 2 | 4 | 103.6 | 14.68 | 5.72 | -9.28 | -1.06 |
| ***41*** | 4-chloroacetanilide | 1.27 | 169.6 | 2.23 | 29.1 | 2.05 | 1 | 2 | 1 | 2 | 134.5 | 18.01 | 3.86 | -8.67 | -0.06 |
| ***42*** | 4-chloroaniline | 1.16 | 127.6 | 2.11 | 26.0 | 1.76 | 1 | 1 | 2 | 1 | 103.7 | 14.03 | 2.74 | -8.11 | 0.38 |
| ***43*** | aniline | 0.35 | 93.1 | 1.97 | 26.0 | 0.94 | 1 | 1 | 2 | 1 | 91.7 | 12.09 | 1.57 | -8.07 | 0.62 |
| ***44*** | antipyrine | 0.22 | 188.2 | 2.27 | 23.6 | 0.27 | 1 | 3 | 0 | 3 | 162.8 | 21.63 | 4.04 | -9.04 | -0.33 |
| ***45*** | benzophenone | 2.06 | 182.2 | 2.26 | 17.1 | 3.18 | 2 | 1 | 0 | 1 | 167.6 | 22.22 | 2.49 | -9.94 | -0.67 |
| ***46*** | benzyl benzoate | 2.71 | 212.3 | 2.33 | 26.3 | 3.97 | 4 | 2 | 0 | 2 | 188.0 | 24.78 | 1.94 | -9.61 | -0.34 |
| ***47*** | bromobenzene | 1.75 | 157.0 | 2.20 | 0.0 | 2.99 | 0 | 0 | 0 | 0 | 105.6 | 13.46 | 1.18 | -9.81 | -0.05 |
| ***48*** | butylbenzene | 2.78 | 134.2 | 2.13 | 0.0 | 4.27 | 3 | 0 | 0 | 0 | 155.3 | 17.87 | 0.22 | -9.51 | 0.36 |
| ***49*** | butyrophenone | 1.56 | 148.2 | 2.17 | 17.1 | 2.73 | 3 | 1 | 0 | 1 | 154.0 | 18.06 | 2.68 | -9.99 | -0.09 |
| ***50*** | caffeine | -0.17 | 194.2 | 2.29 | 53.5 | -0.13 | 0 | 6 | 0 | 6 | 133.4 | 19.97 | 3.87 | -8.89 | -0.49 |
| ***51*** | chlorobenzene | 1.58 | 112.6 | 2.05 | 0.0 | 2.81 | 0 | 0 | 0 | 0 | 101.4 | 12.36 | 0.95 | -9.39 | 0.06 |
| ***52*** | corticosterone | 1.69 | 346.5 | 2.54 | 74.6 | 1.76 | 4 | 4 | 2 | 4 | 284.3 | 37.27 | 2.99 | -10.21 | -0.15 |
| ***53*** | cortisone | 1.29 | 360.5 | 2.56 | 91.7 | 1.44 | 4 | 5 | 2 | 5 | 280.3 | 37.33 | 7.56 | -10.09 | -0.07 |
| ***54*** | estradiol | 2.65 | 272.4 | 2.44 | 40.5 | 4.13 | 2 | 2 | 2 | 2 | 232.6 | 31.52 | 2.64 | -8.86 | -0.35 |
| ***55*** | estriol | 1.66 | 288.4 | 2.46 | 60.7 | 2.94 | 3 | 3 | 3 | 3 | 229.6 | 32.15 | 1.68 | -8.88 | 0.34 |
| ***56*** | ethylbenzene | 1.81 | 106.2 | 2.03 | 0.0 | 3.21 | 1 | 0 | 0 | 0 | 122.3 | 14.19 | 0.22 | -9.51 | 0.38 |
| ***57*** | furan | 0.32 | 68.1 | 1.83 | 13.1 | 1.38 | 0 | 1 | 0 | 1 | 72.2 | 7.35 | 0.22 | -9.38 | 0.61 |
| ***58*** | geraniol | 1.80 | 154.3 | 2.19 | 20.2 | 3.28 | 5 | 1 | 1 | 1 | 177.9 | 19.71 | 1.39 | -9.33 | 0.99 |
| ***59*** | heptanophenone | 2.99 | 190.3 | 2.28 | 17.1 | 4.32 | 6 | 1 | 0 | 1 | 203.5 | 23.57 | 2.63 | -10.00 | -0.42 |
| ***60*** | hydrocortisone | 1.30 | 362.5 | 2.56 | 94.8 | 1.43 | 5 | 5 | 3 | 5 | 281.4 | 37.89 | 3.34 | -10.25 | -0.18 |
| ***61*** | 3-methylphenol | 1.05 | 108.1 | 2.03 | 20.2 | 1.94 | 1 | 1 | 1 | 1 | 104.1 | 13.07 | 1.41 | -9.09 | 0.29 |
| ***62*** | methyl benzoate | 1.11 | 136.2 | 2.13 | 26.3 | 2.20 | 2 | 2 | 0 | 2 | 127.3 | 15.07 | 2.07 | -10.11 | -0.46 |
| ***63*** | monuron | 1.24 | 198.7 | 2.30 | 32.2 | 1.89 | 1 | 3 | 1 | 3 | 158.2 | 21.32 | 2.21 | -9.13 | -0.20 |
| ***64*** | myrcene | 3.01 | 136.2 | 2.13 | 0.0 | 4.58 | 4 | 0 | 0 | 0 | 177.0 | 18.86 | 0.38 | -9.28 | 0.29 |
| ***65*** | o-toluidine | 0.65 | 107.2 | 2.03 | 16.0 | 1.40 | 1 | 1 | 2 | 1 | 108.0 | 14.00 | 1.63 | -7.99 | 0.59 |
| ***66*** | propiophenone | 1.16 | 134.2 | 2.13 | 17.1 | 2.20 | 2 | 1 | 0 | 1 | 137.5 | 16.22 | 2.68 | -9.99 | -0.42 |
| ***67*** | propylbenzene | 2.29 | 120.2 | 2.08 | 0.0 | 3.74 | 2 | 0 | 0 | 0 | 138.8 | 16.03 | 0.22 | -9.51 | 0.37 |
| ***68*** | p-xylene | 1.83 | 106.2 | 2.03 | 0.0 | 3.14 | 0 | 0 | 0 | 0 | 122.0 | 14.23 | 0.00 | -9.18 | 0.36 |
| ***69*** | pyrimidine | -0.50 | 80.1 | 1.90 | 25.8 | -0.33 | 0 | 2 | 0 | 2 | 75.9 | 8.89 | 1.99 | -10.29 | -0.41 |
| ***70*** | pyrocatechol | 0.49 | 110.1 | 2.04 | 40.5 | 0.88 | 2 | 2 | 2 | 2 | 86.3 | 11.90 | 0.59 | -9.07 | 0.26 |
| ***71*** | pyrrole | 0.18 | 67.1 | 1.83 | 15.8 | 0.75 | 0 | 1 | 1 | 1 | 67.8 | 8.20 | 2.18 | -8.93 | 1.11 |
| ***72*** | quinoline | 2.07 | 129.2 | 2.11 | 12.9 | 2.08 | 0 | 1 | 0 | 1 | 116.8 | 16.72 | 1.85 | -9.24 | -0.65 |
| ***73*** | resorcinol | 0.40 | 110.1 | 2.04 | 40.5 | 0.76 | 2 | 2 | 2 | 2 | 86.3 | 11.90 | 1.12 | -9.06 | 0.27 |
| ***74*** | thiourea | -0.77 | 76.1 | 1.88 | 88.7 | -0.39 | 0 | 2 | 3 | 2 | 29.9 | 7.26 | 6.26 | -8.62 | -0.27 |
| ***75*** | thymol | 2.19 | 150.2 | 2.18 | 20.2 | 3.28 | 2 | 1 | 1 | 1 | 154.2 | 18.69 | 1.34 | -9.00 | 0.28 |
| ***76*** | valerophenone | 2.01 | 162.2 | 2.21 | 17.1 | 3.26 | 4 | 1 | 0 | 1 | 170.5 | 19.89 | 2.54 | -9.99 | -0.42 |
| ***77*** | pentane | 2.28 | 72.2 | 1.86 | 0.0 | 3.41 | 2 | 0 | 0 | 0 | 111.1 | 10.00 | 0.00 | -11.30 | 3.45 |
| ***78*** | dichloromethane | 0.22 | 84.9 | 1.93 | 0.0 | 1.19 | 0 | 0 | 0 | 0 | 67.8 | 6.49 | 1.36 | -10.48 | 0.52 |
| ***79*** | chloroform | 0.62 | 119.4 | 2.08 | 0.0 | 1.76 | 0 | 0 | 0 | 0 | 79.6 | 8.40 | 1.02 | -10.88 | -0.12 |
| ***80*** | carbon tetrachloride | 1.61 | 153.8 | 2.19 | 0.0 | 2.86 | 0 | 0 | 0 | 0 | 90.6 | 10.32 | 0.00 | -10.99 | -0.63 |
| ***81*** | 1,2-dichloroethane | 0.34 | 99.0 | 2.00 | 0.0 | 1.41 | 1 | 0 | 0 | 0 | 84.3 | 8.33 | 0.00 | -10.69 | 0.54 |
| ***82*** | 1,1,2,2,-tetrachloroethane | 1.28 | 167.9 | 2.22 | 0.0 | 2.17 | 1 | 0 | 0 | 0 | 107.8 | 12.14 | 1.55 | -10.74 | 0.02 |
| ***83*** | 1-chlorobutane | 1.05 | 92.6 | 1.97 | 0.0 | 2.56 | 2 | 0 | 0 | 0 | 106.0 | 10.08 | 1.56 | -10.31 | 1.23 |
| ***84*** | diethyl ether | -0.06 | 74.1 | 1.87 | 9.2 | 0.98 | 2 | 1 | 0 | 1 | 100.9 | 8.85 | 1.15 | -10.48 | 2.86 |
| ***85*** | dipropyl ether | 0.78 | 102.2 | 2.01 | 9.2 | 2.04 | 4 | 1 | 0 | 1 | 133.9 | 12.52 | 1.11 | -10.49 | 2.76 |
| ***86*** | methyl acetate | -0.66 | 74.1 | 1.87 | 26.3 | 0.18 | 1 | 2 | 0 | 2 | 81.5 | 7.03 | 1.80 | -11.26 | 1.02 |
| ***87*** | ethyl acetate | -0.25 | 88.1 | 1.95 | 26.3 | 0.71 | 2 | 2 | 0 | 2 | 98.0 | 8.83 | 1.88 | -11.24 | 1.06 |
| ***88*** | butyl acetate | 0.62 | 116.2 | 2.07 | 26.3 | 1.77 | 4 | 2 | 0 | 2 | 131.0 | 12.54 | 1.89 | -11.25 | 1.05 |
| ***89*** | acetonitrile | -0.70 | 41.1 | 1.61 | 23.8 | -0.45 | 0 | 1 | 0 | 1 | 54.9 | 4.45 | 3.21 | -12.33 | 1.40 |
| ***90*** | propionitrile | -0.35 | 55.1 | 1.74 | 23.8 | 0.08 | 0 | 1 | 0 | 1 | 71.4 | 6.29 | 3.25 | -12.01 | 1.42 |
| ***91*** | ethanol | -0.62 | 46.1 | 1.66 | 20.2 | -0.19 | 1 | 1 | 1 | 1 | 59.1 | 5.09 | 1.45 | -10.90 | 3.33 |
| ***92*** | 1-propanol | -0.42 | 60.1 | 1.78 | 20.2 | 0.34 | 2 | 1 | 1 | 1 | 75.6 | 6.93 | 1.43 | -10.88 | 3.23 |
| ***93*** | 2-methyl-2-propanol | 0.37 | 74.1 | 1.87 | 20.2 | 0.51 | 1 | 1 | 1 | 1 | 92.1 | 8.75 | 1.54 | -11.28 | 3.26 |
| ***94*** | 1-pentanol | 0.33 | 88.2 | 1.95 | 20.2 | 1.41 | 4 | 1 | 1 | 1 | 108.6 | 10.60 | 1.42 | -10.89 | 3.11 |
| ***95*** | 2-methyl-2-butanol | -0.08 | 88.2 | 1.95 | 20.2 | 1.04 | 2 | 1 | 1 | 1 | 108.6 | 10.59 | 1.64 | -11.13 | 3.17 |
| ***96*** | 1-hexanol | 0.83 | 102.2 | 2.01 | 20.2 | 1.94 | 5 | 1 | 1 | 1 | 125.1 | 12.44 | 1.41 | -10.89 | 3.09 |
| ***97*** | formic acid | -0.65 | 46.0 | 1.66 | 37.3 | -0.54 | 0 | 2 | 1 | 2 | 39.9 | 3.33 | 1.51 | -11.57 | 0.97 |
| ***98*** | acetic acid | -0.68 | 60.1 | 1.78 | 37.3 | -0.29 | 0 | 2 | 1 | 2 | 56.2 | 5.11 | 1.82 | -11.43 | 0.93 |
| ***99*** | propionic acid | -0.08 | 74.1 | 1.87 | 37.3 | 0.25 | 1 | 2 | 1 | 2 | 72.7 | 6.94 | 1.93 | -11.31 | 0.96 |
| ***100*** | butanoic acid | 0.38 | 88.1 | 1.95 | 37.3 | 0.78 | 2 | 2 | 1 | 2 | 89.2 | 8.78 | 1.98 | -11.34 | 0.97 |
| ***101*** | tetrahydrofuran | -0.18 | 72.1 | 1.86 | 9.2 | 0.33 | 0 | 1 | 0 | 1 | 79.8 | 7.95 | 1.66 | -10.28 | 3.29 |
| ***102*** | ethyl benzoate | 1.48 | 150.2 | 2.18 | 26.3 | 2.73 | 3 | 2 | 0 | 2 | 143.8 | 16.91 | 2.08 | -10.08 | -0.43 |
| ***103*** | 2-chloro-1-nitrobenzene | 1.44 | 157.6 | 2.20 | 45.8 | 2.34 | 3 | 1 | 0 | 3 | 113.2 | 14.94 | 5.39 | -9.94 | -1.27 |
| ***104*** | phenylacetonitrile | 0.82 | 117.2 | 2.07 | 23.8 | 1.45 | 1 | 1 | 0 | 1 | 115.7 | 14.16 | 3.30 | -9.99 | -0.09 |
| ***105*** | 2-naphthylamine | 1.77 | 143.2 | 2.16 | 26.0 | 2.17 | 1 | 1 | 2 | 1 | 125.8 | 19.16 | 1.85 | -7.92 | -0.21 |
| ***106*** | 3-chlorophenol | 1.70 | 128.6 | 2.11 | 20.2 | 2.40 | 1 | 1 | 1 | 1 | 99.8 | 13.09 | 0.44 | -9.24 | -0.01 |
| ***107*** | 3-methylbenzoic acid | 1.43 | 136.2 | 2.13 | 37.3 | 2.35 | 1 | 2 | 1 | 2 | 118.2 | 15.07 | 2.51 | -9.82 | -0.49 |
| ***108*** | 4-nitrophenol | 1.28 | 139.1 | 2.14 | 66.1 | 1.57 | 2 | 4 | 1 | 4 | 99.7 | 13.75 | 5.57 | -10.17 | -1.08 |
| ***109*** | 4-chlorobenzyl alcohol | 1.06 | 142.6 | 2.15 | 20.2 | 1.63 | 2 | 1 | 1 | 1 | 115.2 | 14.91 | 2.19 | -9.28 | 0.02 |
| ***110*** | 1,3-dichlorobenzene | 2.48 | 147.0 | 2.17 | 0.0 | 3.42 | 0 | 0 | 0 | 0 | 113.3 | 14.29 | 0.88 | -9.42 | -0.19 |
| ***111*** | 1,3,5-trimethylbenzene | 2.61 | 120.2 | 2.08 | 0.0 | 3.60 | 0 | 0 | 0 | 0 | 138.3 | 16.15 | 0.00 | -9.28 | 0.44 |
| ***112*** | heptane | 3.20 | 100.2 | 2.00 | 0.0 | 4.47 | 4 | 4 | 0 | 0 | 144.1 | 13.67 | 0.00 | -11.27 | 3.30 |
| ***113*** | hexane | 1.97 | 86.2 | 1.94 | 0.0 | 3.94 | 3 | 0 | 0 | 0 | 127.6 | 11.83 | 0.00 | -11.28 | 3.36 |
| ***114*** | acetaminophen | 0.38 | 151.2 | 2.18 | 49.3 | 1.66 | 1 | 1 | 2 | 3 | 121.0 | 16.81 | 2.72 | -8.56 | -0.02 |
| ***115*** | nimodipine | 3.06 | 418.5 | 2.62 | 119.7 | 3.85 | 10 | 9 | 1 | 9 | 345.0 | 42.87 | 10.27 | -9.21 | -0.66 |
| ***116*** | progesterone | 2.98 | 314.5 | 2.50 | 34.1 | 4.04 | 1 | 2 | 0 | 2 | 289.0 | 36.06 | 4.82 | -10.14 | -0.10 |
| ***117*** | tinidazole | 0.26 | 247.3 | 2.39 | 101.2 | -0.27 | 5 | 7 | 0 | 7 | 172.4 | 23.36 | 2.19 | -10.47 | -1.21 |
| ***118*** | p-toluidine | 1.15 | 107.2 | 2.03 | 26.0 | 1.40 | 1 | 1 | 2 | 1 | 108.0 | 14.00 | 1.38 | -7.95 | 0.64 |
| ***119*** | 2-aminobiphenyl | 2.13 | 169.2 | 2.23 | 26.0 | 2.68 | 2 | 1 | 2 | 1 | 157.0 | 21.84 | 1.42 | -7.98 | -0.21 |
| ***120*** | N-ethylaniline | 1.06 | 121.2 | 2.08 | 12.0 | 2.13 | 2 | 1 | 1 | 1 | 125.4 | 16.05 | 1.36 | -8.50 | 0.45 |
| ***121*** | 1-naphthoic acid | 2.13 | 172.2 | 2.24 | 37.3 | 3.13 | 1 | 2 | 1 | 2 | 136.1 | 20.23 | 2.26 | -9.13 | -0.97 |
| ***122*** | phenylacetic acid | 0.76 | 136.2 | 2.13 | 37.3 | 1.50 | 2 | 2 | 1 | 2 | 116.9 | 14.81 | 1.83 | -9.81 | 0.22 |
| ***123*** | 3-chlorophenylacetic acid | 1.47 | 170.6 | 2.23 | 37.3 | 2.10 | 2 | 2 | 1 | 2 | 128.8 | 16.75 | 1.23 | -9.52 | -0.20 |
| ***124*** | 4-phenylbutanoic acid | 1.52 | 150.2 | 2.18 | 37.3 | 2.42 | 4 | 2 | 1 | 2 | 149.9 | 18.49 | 1.82 | -9.67 | 0.21 |
| ***125*** | 1,2-diphenylethane | 3.77 | 182.3 | 2.26 | 0.0 | 4.70 | 2 | 0 | 0 | 0 | 183.0 | 23.90 | 0.00 | -9.48 | 0.32 |
| ***126*** | testosterone | 2.23 | 288.4 | 2.46 | 37.3 | 3.48 | 1 | 2 | 1 | 2 | 257.0 | 32.95 | 4.22 | -10.12 | -0.09 |
| ***127*** | 2-butanone | -0.38 | 72.1 | 1.86 | 17.1 | 0.37 | 1 | 1 | 0 | 1 | 91.7 | 8.17 | 2.56 | -10.65 | 0.83 |
| ***128*** | theophylline | 0.15 | 180.2 | 2.26 | 69.3 | -0.17 | 0 | 6 | 1 | 6 | 122.9 | 17.11 | 3.48 | -8.98 | -0.53 |
| ***129*** | pyridine | 0.32 | 79.1 | 1.90 | 12.9 | 0.73 | 0 | 1 | 0 | 1 | 82.7 | 9.65 | 1.94 | -10.10 | 0.01 |
| ***130*** | 4-ethylphenol | 1.54 | 122.2 | 2.09 | 20.2 | 2.47 | 2 | 1 | 1 | 1 | 120.7 | 14.94 | 1.16 | -9.00 | 0.32 |
| ***131*** | 4-propylphenol | 2.02 | 136.2 | 2.13 | 20.2 | 3.00 | 3 | 1 | 1 | 1 | 137.2 | 16.78 | 1.16 | -9.00 | 0.32 |
| ***132*** | 4-butylphenol | 2.51 | 150.2 | 2.18 | 20.2 | 3.54 | 4 | 1 | 1 | 1 | 153.7 | 18.61 | 1.14 | -8.99 | 0.32 |
| ***133*** | 4-fluorophenol | 0.96 | 112.1 | 2.05 | 20.2 | 1.77 | 1 | 1 | 1 | 1 | 92.1 | 11.15 | 1.84 | -9.27 | -0.06 |
| ***134*** | 4-bromophenol | 1.81 | 173.0 | 2.24 | 20.2 | 2.49 | 1 | 1 | 1 | 1 | 104.1 | 14.20 | 1.52 | -9.31 | -0.03 |
| ***135*** | 4-iodophenol | 2.10 | 220.0 | 2.34 | 20.2 | 2.91 | 1 | 1 | 1 | 1 | 109.9 | 16.27 | 1.28 | -8.84 | -0.41 |
| ***136*** | benzyl methyl ketone | 0.38 | 134.2 | 2.13 | 17.1 | 1.44 | 2 | 1 | 0 | 1 | 135.9 | 16.04 | 0.00 | -9.71 | 0.07 |
| ***137*** | isradipine | 2.48 | 371.4 | 2.57 | 103.6 | 3.59 | 6 | 8 | 1 | 8 | 297.2 | 38.42 | 6.32 | -8.84 | -1.45 |
| ***138*** | felodipine | 3.47 | 384.3 | 2.58 | 64.6 | 4.83 | 6 | 5 | 1 | 5 | 300.8 | 37.97 | 5.31 | -8.87 | -0.18 |
| ***139*** | nisoldipine | 3.26 | 388.4 | 2.59 | 110.5 | 4.38 | 8 | 8 | 1 | 8 | 322.2 | 40.34 | 6.27 | -9.15 | -0.73 |
| ***140*** | lacidipine | 4.00 | 455.6 | 2.66 | 90.9 | 5.48 | 11 | 7 | 1 | 7 | 404.0 | 50.26 | 1.40 | -8.91 | -1.29 |
| ***141*** | trimethoprin | 0.95 | 290.3 | 2.46 | 105.5 | 0.79 | 5 | 7 | 4 | 7 | 231.9 | 31.82 | 2.19 | -8.73 | -0.07 |
| ***142*** | prednisolone | 1.65 | 360.5 | 2.56 | 94.8 | 1.49 | 5 | 3 | 3 | 5 | 274.7 | 37.85 | 3.75 | -10.07 | -0.42 |
| ***143*** | acetylsalicylic acid | 0.82 | 180.2 | 2.26 | 63.6 | 1.19 | 3 | 4 | 1 | 4 | 139.6 | 17.65 | 4.18 | -10.19 | -0.54 |
| ***144*** | benzoic acid | 1.05 | 122.1 | 2.09 | 37.3 | 1.89 | 1 | 2 | 1 | 2 | 102.0 | 13.15 | 2.26 | -10.13 | -0.53 |
| ***145*** | pramocaine | 2.56 | 293.4 | 2.47 | 30.9 | 3.48 | 9 | 4 | 0 | 4 | 284.6 | 33.45 | 2.52 | -8.78 | 0.24 |
| ***146*** | acetone | -0.75 | 58.1 | 1.76 | 17.1 | -0.16 | 0 | 1 | 0 | 1 | 75.2 | 6.33 | 2.73 | -10.76 | 0.80 |
| ***147*** | 4-aminophenol | -0.20 | 109.1 | 2.04 | 46.3 | -0.29 | 2 | 2 | 3 | 2 | 90.1 | 12.83 | 2.09 | -7.84 | 0.46 |
| ***148*** | methyl nicotinate | 0.27 | 137.1 | 2.14 | 39.2 | 0.88 | 2 | 3 | 0 | 3 | 120.6 | 14.32 | 0.47 | -10.44 | -0.80 |
| ***149*** | N,N-dimethylformamide | -0.47 | 73.1 | 1.86 | 20.3 | -1.01 | 0 | 2 | 0 | 2 | 82.6 | 7.87 | 3.46 | -9.26 | 1.36 |
| ***150*** | ketoprofen | 0.93 | 254.3 | 2.41 | 54.4 | 2.81 | 4 | 3 | 1 | 3 | 212.3 | 28.46 | 0.89 | -9.97 | -0.40 |
| ***151*** | naproxen | 0.99 | 230.3 | 2.36 | 46.5 | 3.00 | 3 | 3 | 1 | 3 | 192.3 | 26.37 | 2.42 | -8.67 | -0.53 |
| ***152*** | ibuprofen | 1.35 | 206.3 | 2.31 | 37.3 | 3.72 | 4 | 2 | 1 | 2 | 200.3 | 24.09 | 1.86 | -9.51 | 0.06 |
| ***153*** | salicylic acid | 0.08 | 138.1 | 2.14 | 57.5 | 2.06 | 3 | 3 | 2 | 3 | 100.4 | 13.90 | 0.99 | -9.46 | -0.60 |
| ***154*** | diclofenac | 2.21 | 296.2 | 2.47 | 49.3 | 4.06 | 4 | 3 | 2 | 3 | 206.8 | 30.34 | 5.14 | -8.27 | -1.38 |
| ***155*** | captopril | -0.79 | 217.3 | 2.34 | 96.4 | 0.27 | 4 | 4 | 1 | 4 | 170.7 | 21.58 | 2.68 | -9.34 | 0.11 |
| ***156*** | flurbiprofen | 1.54 | 244.3 | 2.39 | 37.3 | 4.12 | 3 | 2 | 1 | 2 | 103.6 | 26.40 | 1.98 | -9.07 | -0.70 |
| ***157*** | indomethacin | 2.19 | 357.8 | 2.55 | 63.6 | 3.10 | 4 | 5 | 1 | 5 | 269.6 | 37.50 | 2.36 | -8.56 | -0.61 |
| ***158*** | decanoic acid | 0.84 | 172.3 | 2.24 | 37.3 | 3.97 | 8 | 2 | 1 | 2 | 188.2 | 19.80 | 2.05 | -11.37 | 0.96 |
| ***159*** | 3-nitrobenzoic acid | -0.30 | 167.1 | 2.22 | 83.1 | 1.82 | 2 | 5 | 1 | 5 | 113.8 | 15.75 | 3.32 | -10.95 | -1.49 |
| ***160*** | procaine | 1.02 | 236.3 | 2.37 | 55.6 | 2.36 | 8 | 4 | 2 | 4 | 219.4 | 27.41 | 3.37 | -8.92 | -0.37 |
| ***161*** | lidocaine | 1.09 | 234.3 | 2.37 | 32.3 | 2.36 | 5 | 3 | 1 | 3 | 228.4 | 28.71 | 3.75 | -9.15 | -0.06 |
| ***162*** | ropivacaine | 1.54 | 274.4 | 2.44 | 32.3 | 3.11 | 4 | 3 | 1 | 3 | 262.7 | 33.30 | 2.21 | -8.88 | 0.10 |
| ***163*** | timolol | 0.90 | 316.4 | 2.50 | 108.0 | 0.06 | 8 | 7 | 2 | 7 | 258.5 | 32.57 | 2.14 | -9.24 | -1.05 |
| ***164*** | bupivacaine | 1.88 | 288.4 | 2.46 | 32.3 | 3.64 | 5 | 3 | 1 | 3 | 279.2 | 35.13 | 7.37 | -7.46 | -0.40 |
| ***165*** | prilocaine | 0.99 | 220.3 | 2.34 | 41.1 | 1.74 | 5 | 3 | 2 | 3 | 214.0 | 26.73 | 2.89 | -9.23 | 0.18 |
| ***166*** | atenolol | 0.65 | 266.3 | 2.43 | 84.6 | 0.10 | 9 | 5 | 2 | 5 | 236.7 | 29.44 | 3.50 | -9.30 | -0.03 |
| ***167*** | propranolol | 2.20 | 259.4 | 2.41 | 41.5 | 3.10 | 7 | 3 | 2 | 3 | 237.2 | 31.31 | 1.26 | -8.62 | -0.43 |
| ***168*** | pseudoefedrine | 0.36 | 165.2 | 2.22 | 32.3 | 1.05 | 4 | 2 | 2 | 2 | 162.7 | 19.88 | 1.19 | -9.44 | 0.29 |
| ***169*** | acetbutolol | 1.57 | 336.4 | 2.53 | 87.7 | 1.95 | 11 | 6 | 3 | 6 | 300.7 | 37.55 | 4.38 | -9.15 | -0.43 |
| ***170*** | alprenolol | 2.08 | 249.4 | 2.40 | 41.5 | 2.88 | 9 | 3 | 2 | 3 | 247.5 | 29.75 | 1.40 | -9.12 | 0.26 |
| ***171*** | metoprolol | 1.21 | 267.4 | 2.43 | 50.7 | 1.79 | 10 | 4 | 2 | 4 | 258.7 | 30.55 | 1.83 | -9.00 | -0.27 |
| ***172*** | oxprenolol | 1.55 | 265.4 | 2.42 | 50.7 | 2.29 | 10 | 4 | 2 | 4 | 255.2 | 30.45 | 2.81 | -9.21 | 0.12 |
| ***173*** | albuterol | 0.48 | 239.3 | 2.38 | 72.7 | 0.01 | 8 | 4 | 4 | 4 | 207.6 | 26.86 | 2.20 | -8.97 | 0.16 |
| ***174*** | fluoxetine | 2.98 | 309.3 | 2.49 | 21.3 | 4.09 | 6 | 2 | 1 | 2 | 266.7 | 31.67 | 4.92 | -9.44 | -0.39 |
| ***175*** | verapamil | 2.76 | 454.6 | 2.66 | 64.0 | 3.90 | 13 | 6 | 0 | 6 | 429.4 | 52.28 | 3.64 | -8.78 | -0.95 |
