# Supplementary material for: Immobilized artificial membrane-chromatographic and computational descriptors in studies of soil-water partition of environmentally relevant compounds
Source: Environ Sci Pollut Res Int. 2022 Aug 22;30(3):6192–200. doi: 10.1007/s11356-022-22514-x (PMC9895004; doi:10.1007/s11356-022-22514-x)
Supplement: Supplementary file 4 — (DOCX 19 kb) [file 11356_2022_22514_MOESM4_ESM.docx]

Table 4. Properties of ANN’s analyzed In this study

| Index | Net. name | Training perf. - R | Test perf. - R | Validation perf.- R | Training error | Test error | Validation error | Training algorithm | Error function | Hidden activation | Output activation | log ***K_oc_***^pred^ vs. log ***K_oc_***^(4)^ - R^2^ (n=175) | log ***K_oc_***^pred^ vs. log ***K_oc_***^exp^ - R^2^ (n=50) |
| --- | --- | --- | --- | --- | --- | --- | --- | --- | --- | --- | --- | --- | --- |
| ANN_1-1_ | MLP 10-12-1 | 0.97 | 0.97 | 0.97 | 0.030 | 0.010 | 0.019 | BFGS 70 | SOS | Logistic | Logistic | 0.94 | 0.84 |
| ANN_1-2_ | MLP 10-11-1 | 0.97 | 0.97 | 0.98 | 0.027 | 0.012 | 0.018 | BFGS 103 | SOS | Logistic | Exponential | 0.95 | 0.83 |
| ANN_1-3_ | MLP 10-9-1 | 0.96 | 0.97 | 0.97 | 0.040 | 0.013 | 0.019 | BFGS 50 | SOS | Logistic | Exponential | 0.93 | 0.84 |
| ANN_1-4_ | MLP 10-4-1 | 0.96 | 0.97 | 0.98 | 0.038 | 0.012 | 0.017 | BFGS 63 | SOS | Logistic | Exponential | 0.93 | 0.86 |
| ANN_1-5_ | MLP 10-12-1 | 0.97 | 0.97 | 0.97 | 0.031 | 0.011 | 0.020 | BFGS 65 | SOS | Logistic | Logistic | 0.94 | 0.83 |
| ANN_2-1_ | MLP 6-7-1 | 0.97 | 0.98 | 0.97 | 0.032 | 0.009 | 0.023 | BFGS 104 | SOS | Logistic | Tanh | 0.94 | 0.83 |
| ANN_2-2_ | MLP 6-7-1 | 0.98 | 0.98 | 0.97 | 0.024 | 0.009 | 0.031 | BFGS 126 | SOS | Tanh | Exponential | 0.95 | 0.86 |
| ANN_2-3_ | MLP 6-8-1 | 0.96 | 0.98 | 0.97 | 0.036 | 0.009 | 0.023 | BFGS 131 | SOS | Logistic | Exponential | 0.93 | 0.80 |
| ANN_2-4_ | MLP 6-9-1 | 0.97 | 0.98 | 0.97 | 0.030 | 0.009 | 0.024 | BFGS 89 | SOS | Tanh | Exponential | 0.94 | 0.82 |
| ANN_2-5_ | MLP 6-4-1 | 0.95 | 0.97 | 0.97 | 0.045 | 0.012 | 0.022 | BFGS 66 | SOS | Tanh | Tanh | 0.92 | 0.80 |
| ANN_3-1_ | MLP 3-9-1 | 0.95 | 0.94 | 0.98 | 0.047 | 0.022 | 0.017 | BFGS 83 | SOS | Tanh | Logistic | 0.91 | 0.77 |
| ANN_3-2_ | MLP 3-9-1 | 0.95 | 0.96 | 0.98 | 0.049 | 0.015 | 0.016 | BFGS 91 | SOS | Tanh | Tanh | 0.91 | 0.80 |
| ANN_3-3_ | MLP 3-8-1 | 0.95 | 0.94 | 0.98 | 0.048 | 0.022 | 0.017 | BFGS 95 | SOS | Logistic | Tanh | 0.91 | 0.77 |
| ANN_3-4_ | MLP 3-6-1 | 0.95 | 0.95 | 0.97 | 0.050 | 0.020 | 0.018 | BFGS 112 | SOS | Logistic | Tanh | 0.91 | 0.78 |
| ANN_3-5_ | MLP 3-6-1 | 0.95 | 0.95 | 0.98 | 0.049 | 0.018 | 0.014 | BFGS 160 | SOS | Logistic | Tanh | 0.91 | 0.79 |
| ANN_4-1_ | MLP 2-4-1 | 0.93 | 0.95 | 0.97 | 0.064 | 0.021 | 0.025 | BFGS 11 | SOS | Exponential | Logistic | 0.88 | 0.80 |
| ANN_4-2_ | MLP 2-9-1 | 0.94 | 0.95 | 0.97 | 0.063 | 0.020 | 0.023 | BFGS 5 | SOS | Exponential | Logistic | 0.88 | 0.81 |
| ANN_4-3_ | MLP 2-5-1 | 0.93 | 0.95 | 0.97 | 0.074 | 0.020 | 0.023 | BFGS 9 | SOS | Tanh | Tanh | 0.87 | 0.81 |
| ANN_4-4_ | MLP 2-6-1 | 0.94 | 0.95 | 0.97 | 0.061 | 0.020 | 0.024 | BFGS 35 | SOS | Logistic | Logistic | 0.89 | 0.80 |
| ANN_4-5_ | MLP 2-4-1 | 0.94 | 0.95 | 0.97 | 0.060 | 0.020 | 0.025 | BFGS 29 | SOS | Tanh | Logistic | 0.89 | 0.80 |
| MLR Eq. **(6)** |  |  |  |  |  |  |  |  |  |  |  | 0.91 | 0.85 |
| MLR Eq. **(8)** |  |  |  |  |  |  |  |  |  |  |  | 0.91 | 0.83 |
| MLR Eq. **(9)** |  |  |  |  |  |  |  |  |  |  |  | 0.89 | 0.80 |
| MLR Eq. **(10)** |  |  |  |  |  |  |  |  |  |  |  | 0.88 | 0.80 |
