# Supplementary material for: Immobilized artificial membrane-chromatographic and computational descriptors in studies of soil-water partition of environmentally relevant compounds
Source: Environ Sci Pollut Res Int. 2022 Aug 22;30(3):6192–200. doi: 10.1007/s11356-022-22514-x (PMC9895004; doi:10.1007/s11356-022-22514-x)
Supplement: Supplementary file 5 — (DOCX 12 kb) [file 11356_2022_22514_MOESM5_ESM.docx]

Table 5. Clusters formed for log ***K_oc_*** values calculated according to 25 methods (175 compounds)

|  | VARIABLE NO. | CLUSTER NO. | DISTANCE FROM CLUSTER CENTER |
| --- | --- | --- | --- |
| log Koc(4) | 1 | 5 | 0.17 |
| ANN1-1 | 2 | 3 | 0.07 |
| ANN1-2 | 3 | 3 | 0.09 |
| ANN1-3 | 4 | 3 | 0.08 |
| ANN1-4 | 5 | 3 | 0.07 |
| ANN1-5 | 6 | 3 | 0.07 |
| ANN2-1 | 7 | 5 | 0.11 |
| ANN2-2 | 8 | 5 | 0.11 |
| ANN2-3 | 9 | 5 | 0.11 |
| ANN2-4 | 10 | 5 | 0.10 |
| ANN2-5 | 11 | 1 | 0.10 |
| ANN3-1 | 12 | 2 | 0.07 |
| ANN3-2 | 13 | 2 | 0.05 |
| ANN3-3 | 14 | 2 | 0.05 |
| ANN3-4 | 15 | 2 | 0.06 |
| ANN3-5 | 16 | 2 | 0.07 |
| ANN4-1 | 17 | 4 | 0.06 |
| ANN4-2 | 18 | 4 | 0.04 |
| ANN4-3 | 19 | 4 | 0.12 |
| ANN4-4 | 20 | 4 | 0.04 |
| ANN3-5 | 21 | 4 | 0.04 |
| log Koc(6) | 22 | 1 | 0.07 |
| log Koc(8) | 23 | 1 | 0.05 |
| log Koc(9) | 24 | 4 | 0.06 |
| log Koc(10) | 25 | 4 | 0.04 |
