# Supplementary material for: Immobilized artificial membrane-chromatographic and computational descriptors in studies of soil-water partition of environmentally relevant compounds
Source: Environ Sci Pollut Res Int. 2022 Aug 22;30(3):6192–200. doi: 10.1007/s11356-022-22514-x (PMC9895004; doi:10.1007/s11356-022-22514-x)
Supplement: Supplementary file 6 — (DOCX 32 kb) [file 11356_2022_22514_MOESM6_ESM.docx]

Table 6. Mean values of log ***K_oc_*** for clusters **1** to **5**.

| Case ID |  |  |  |  |  |  |
| --- | --- | --- | --- | --- | --- | --- |
|  | Mean Cluster 1 | Mean Cluster 2 | Mean Cluster 3 | Mean Cluster 4 | Mean Cluster 5 | S.D. |
| ***1*** | 1.57 | 1.52 | 1.57 | 1.55 | 1.50 | 0.03 |
| ***2*** | 1.88 | 1.62 | 1.87 | 1.68 | 1.84 | 0.11 |
| ***3*** | 1.15 | 1.19 | 1.05 | 1.28 | 1.09 | 0.08 |
| ***4*** | 1.76 | 1.74 | 1.77 | 1.81 | 1.87 | 0.05 |
| ***5*** | 1.76 | 1.45 | 1.70 | 1.52 | 1.71 | 0.12 |
| ***6*** | 1.37 | 1.16 | 1.23 | 1.24 | 1.30 | 0.07 |
| ***7*** | 1.65 | 1.75 | 1.83 | 1.57 | 1.82 | 0.10 |
| ***8*** | 1.86 | 1.52 | 1.88 | 1.59 | 1.77 | 0.14 |
| ***9*** | 1.35 | 1.28 | 1.33 | 1.34 | 1.35 | 0.03 |
| ***10*** | 3.33 | 3.08 | 3.36 | 3.15 | 3.38 | 0.12 |
| ***11*** | 3.52 | 3.55 | 3.60 | 3.39 | 3.67 | 0.09 |
| ***12*** | 2.38 | 2.60 | 2.31 | 2.50 | 2.31 | 0.11 |
| ***13*** | 2.04 | 1.88 | 2.11 | 1.91 | 2.05 | 0.09 |
| ***14*** | 2.14 | 1.93 | 2.09 | 1.93 | 2.19 | 0.11 |
| ***15*** | 1.08 | 1.03 | 1.09 | 1.08 | 1.03 | 0.03 |
| ***16*** | 2.59 | 2.30 | 2.53 | 2.61 | 2.62 | 0.12 |
| ***17*** | 1.96 | 1.95 | 2.00 | 2.00 | 2.01 | 0.03 |
| ***18*** | 1.19 | 1.18 | 1.16 | 1.26 | 0.95 | 0.11 |
| ***19*** | 1.76 | 1.71 | 1.76 | 1.78 | 1.86 | 0.05 |
| ***20*** | 1.74 | 1.71 | 1.77 | 1.78 | 1.85 | 0.05 |
| ***21*** | 2.98 | 2.71 | 3.00 | 2.77 | 3.00 | 0.13 |
| ***22*** | 2.70 | 2.56 | 2.72 | 2.61 | 2.69 | 0.06 |
| ***23*** | 2.64 | 2.49 | 2.64 | 2.52 | 2.64 | 0.07 |
| ***24*** | 1.92 | 2.09 | 2.16 | 1.96 | 2.17 | 0.10 |
| ***25*** | 1.87 | 2.04 | 2.06 | 1.89 | 2.06 | 0.09 |
| ***26*** | 1.99 | 1.95 | 1.94 | 1.85 | 1.93 | 0.04 |
| ***27*** | 1.82 | 1.86 | 1.94 | 1.77 | 1.91 | 0.06 |
| ***28*** | 1.28 | 1.45 | 1.30 | 1.34 | 1.32 | 0.06 |
| ***29*** | 2.23 | 2.15 | 2.17 | 2.11 | 2.15 | 0.04 |
| ***30*** | 1.49 | 1.40 | 1.47 | 1.48 | 1.59 | 0.06 |
| ***31*** | 1.56 | 1.48 | 1.58 | 1.54 | 1.54 | 0.03 |
| ***32*** | 3.09 | 3.01 | 3.11 | 3.02 | 3.05 | 0.04 |
| ***33*** | 1.96 | 2.04 | 2.09 | 1.94 | 2.12 | 0.07 |
| ***34*** | 2.11 | 2.13 | 2.07 | 2.17 | 2.13 | 0.03 |
| ***35*** | 2.19 | 2.06 | 2.18 | 2.13 | 2.22 | 0.06 |
| ***36*** | 2.06 | 2.06 | 2.06 | 2.11 | 2.09 | 0.02 |
| ***37*** | 2.07 | 2.07 | 2.09 | 2.12 | 2.09 | 0.02 |
| ***38*** | 1.96 | 2.02 | 1.99 | 2.00 | 2.05 | 0.03 |
| ***39*** | 1.91 | 1.96 | 1.98 | 1.99 | 2.07 | 0.05 |
| ***40*** | 1.87 | 2.01 | 2.03 | 1.84 | 2.10 | 0.10 |
| ***41*** | 2.13 | 2.25 | 2.19 | 2.20 | 2.13 | 0.04 |
| ***42*** | 1.88 | 1.92 | 1.94 | 1.96 | 2.05 | 0.06 |
| ***43*** | 1.21 | 1.18 | 1.21 | 1.24 | 1.34 | 0.05 |
| ***44*** | 1.62 | 1.63 | 1.34 | 1.58 | 1.31 | 0.14 |
| ***45*** | 3.02 | 2.88 | 3.05 | 2.76 | 3.08 | 0.12 |
| ***46*** | 3.32 | 3.25 | 3.26 | 3.27 | 3.24 | 0.03 |
| ***47*** | 2.47 | 2.60 | 2.44 | 2.47 | 2.45 | 0.06 |
| ***48*** | 3.03 | 2.88 | 2.90 | 2.99 | 2.90 | 0.06 |
| ***49*** | 2.41 | 2.31 | 2.41 | 2.30 | 2.32 | 0.05 |
| ***50*** | 1.06 | 1.17 | 0.90 | 1.31 | 1.15 | 0.14 |
| ***51*** | 2.21 | 2.24 | 2.24 | 2.16 | 2.28 | 0.04 |
| ***52*** | 3.21 | 3.17 | 3.40 | 2.98 | 3.35 | 0.15 |
| ***53*** | 2.92 | 2.85 | 3.12 | 2.73 | 2.90 | 0.12 |
| ***54*** | 3.53 | 3.63 | 3.72 | 3.42 | 3.68 | 0.11 |
| ***55*** | 2.80 | 3.07 | 3.01 | 2.82 | 2.93 | 0.10 |
| ***56*** | 2.29 | 2.29 | 2.31 | 2.26 | 2.36 | 0.03 |
| ***57*** | 1.04 | 0.96 | 1.12 | 1.01 | 1.20 | 0.09 |
| ***58*** | 2.36 | 2.50 | 2.34 | 2.48 | 2.20 | 0.11 |
| ***59*** | 3.54 | 3.35 | 3.44 | 3.36 | 3.36 | 0.07 |
| ***60*** | 2.92 | 2.87 | 2.88 | 2.74 | 3.02 | 0.09 |
| ***61*** | 1.77 | 1.73 | 1.77 | 1.80 | 1.84 | 0.04 |
| ***62*** | 2.00 | 1.95 | 1.91 | 1.96 | 1.89 | 0.04 |
| ***63*** | 2.14 | 2.26 | 1.96 | 2.28 | 2.16 | 0.11 |
| ***64*** | 3.20 | 2.97 | 3.09 | 3.14 | 3.03 | 0.08 |
| ***65*** | 1.46 | 1.47 | 1.37 | 1.53 | 1.60 | 0.08 |
| ***66*** | 2.18 | 1.97 | 2.17 | 1.99 | 2.11 | 0.09 |
| ***67*** | 2.65 | 2.58 | 2.59 | 2.62 | 2.62 | 0.02 |
| ***68*** | 2.31 | 2.30 | 2.39 | 2.27 | 2.39 | 0.05 |
| ***69*** | 0.77 | 0.38 | 0.65 | 0.49 | 0.61 | 0.13 |
| ***70*** | 1.33 | 1.36 | 1.34 | 1.44 | 1.34 | 0.04 |
| ***71*** | 0.83 | 0.87 | 0.76 | 0.88 | 0.98 | 0.07 |
| ***72*** | 2.71 | 2.44 | 2.57 | 2.52 | 2.52 | 0.09 |
| ***73*** | 1.28 | 1.29 | 1.27 | 1.37 | 1.22 | 0.05 |
| ***74*** | 0.30 | 0.18 | 0.33 | 0.28 | 0.21 | 0.06 |
| ***75*** | 2.65 | 2.67 | 2.60 | 2.71 | 2.52 | 0.07 |
| ***76*** | 2.82 | 2.67 | 2.78 | 2.65 | 2.72 | 0.07 |
| ***77*** | 1.98 | 2.11 | 2.03 | 2.32 | 1.96 | 0.13 |
| ***78*** | 1.14 | 1.39 | 1.34 | 1.09 | 1.27 | 0.11 |
| ***79*** | 1.64 | 1.76 | 1.68 | 1.59 | 1.78 | 0.07 |
| ***80*** | 2.43 | 2.51 | 2.18 | 2.37 | 2.29 | 0.11 |
| ***81*** | 1.29 | 1.51 | 1.60 | 1.28 | 1.48 | 0.13 |
| ***82*** | 2.22 | 2.44 | 2.12 | 2.22 | 2.25 | 0.10 |
| ***83*** | 1.56 | 1.88 | 1.74 | 1.72 | 1.73 | 0.10 |
| ***84*** | 0.59 | 0.79 | 0.73 | 0.77 | 0.63 | 0.08 |
| ***85*** | 1.26 | 1.52 | 1.33 | 1.60 | 1.24 | 0.14 |
| ***86*** | 0.39 | 0.23 | 0.39 | 0.33 | 0.43 | 0.07 |
| ***87*** | 0.71 | 0.62 | 0.65 | 0.74 | 0.68 | 0.04 |
| ***88*** | 1.38 | 1.50 | 1.32 | 1.56 | 1.45 | 0.08 |
| ***89*** | 0.24 | 0.37 | 0.38 | 0.04 | 0.41 | 0.14 |
| ***90*** | 0.56 | 0.53 | 0.52 | 0.34 | 0.55 | 0.08 |
| ***91*** | 0.03 | 0.40 | 0.30 | 0.11 | 0.25 | 0.13 |
| ***92*** | 0.29 | 0.51 | 0.35 | 0.35 | 0.39 | 0.08 |
| ***93*** | 0.83 | 1.04 | 0.67 | 1.10 | 0.61 | 0.20 |
| ***94*** | 0.91 | 1.13 | 0.77 | 1.19 | 0.87 | 0.16 |
| ***95*** | 0.73 | 0.86 | 0.54 | 0.88 | 0.67 | 0.13 |
| ***96*** | 1.30 | 1.55 | 1.14 | 1.63 | 1.29 | 0.18 |
| ***97*** | 0.15 | 0.03 | 0.22 | 0.09 | 0.16 | 0.06 |
| ***98*** | 0.27 | 0.11 | 0.26 | 0.19 | 0.28 | 0.06 |
| ***99*** | 0.70 | 0.60 | 0.57 | 0.74 | 0.52 | 0.08 |
| ***100*** | 1.05 | 1.07 | 0.95 | 1.22 | 0.89 | 0.11 |
| ***101*** | 0.46 | 0.70 | 0.60 | 0.65 | 0.52 | 0.09 |
| ***102*** | 2.28 | 2.25 | 2.16 | 2.25 | 2.14 | 0.06 |
| ***103*** | 2.51 | 2.32 | 2.20 | 2.27 | 2.59 | 0.15 |
| ***104*** | 1.84 | 1.64 | 1.98 | 1.70 | 1.78 | 0.12 |
| ***105*** | 2.51 | 2.39 | 2.62 | 2.41 | 2.60 | 0.09 |
| ***106*** | 2.22 | 2.25 | 2.11 | 2.30 | 2.21 | 0.06 |
| ***107*** | 2.18 | 2.14 | 2.15 | 2.16 | 2.02 | 0.06 |
| ***108*** | 2.04 | 2.15 | 2.14 | 2.07 | 2.09 | 0.04 |
| ***109*** | 1.96 | 1.97 | 1.98 | 1.97 | 1.93 | 0.02 |
| ***110*** | 2.90 | 2.86 | 2.78 | 2.87 | 2.78 | 0.05 |
| ***111*** | 2.81 | 2.70 | 2.73 | 2.81 | 2.70 | 0.05 |
| ***112*** | 2.51 | 2.49 | 2.38 | 2.99 | 2.52 | 0.21 |
| ***113*** | 1.97 | 2.20 | 2.09 | 2.23 | 2.09 | 0.09 |
| ***114*** | 1.59 | 1.57 | 1.35 | 1.56 | 1.64 | 0.10 |
| ***115*** | 3.68 | 3.98 | 3.69 | 3.90 | 3.86 | 0.12 |
| ***116*** | 3.99 | 3.94 | 4.14 | 3.72 | 3.88 | 0.14 |
| ***117*** | 1.63 | 1.23 | 1.81 | 1.74 | 1.64 | 0.20 |
| ***118*** | 1.76 | 1.78 | 1.84 | 1.86 | 1.98 | 0.08 |
| ***119*** | 2.83 | 2.80 | 2.98 | 2.75 | 3.01 | 0.10 |
| ***120*** | 1.83 | 1.82 | 1.81 | 1.87 | 1.98 | 0.06 |
| ***121*** | 2.90 | 2.72 | 2.96 | 2.76 | 2.89 | 0.09 |
| ***122*** | 1.64 | 1.72 | 1.75 | 1.74 | 1.53 | 0.08 |
| ***123*** | 2.25 | 2.37 | 2.34 | 2.33 | 2.07 | 0.11 |
| ***124*** | 2.20 | 2.27 | 2.27 | 2.28 | 2.04 | 0.09 |
| ***125*** | 3.87 | 3.62 | 3.73 | 3.78 | 3.68 | 0.09 |
| ***126*** | 3.47 | 3.58 | 3.56 | 3.21 | 3.44 | 0.13 |
| ***127*** | 0.67 | 0.59 | 0.64 | 0.50 | 0.59 | 0.06 |
| ***128*** | 1.17 | 1.38 | 1.14 | 1.48 | 1.06 | 0.16 |
| ***129*** | 1.29 | 1.05 | 1.19 | 1.11 | 1.21 | 0.08 |
| ***130*** | 2.10 | 2.11 | 2.09 | 2.17 | 2.11 | 0.03 |
| ***131*** | 2.45 | 2.47 | 2.38 | 2.53 | 2.36 | 0.06 |
| ***132*** | 2.81 | 2.82 | 2.69 | 2.90 | 2.63 | 0.10 |
| ***133*** | 1.77 | 1.70 | 1.70 | 1.76 | 1.79 | 0.04 |
| ***134*** | 2.42 | 2.66 | 2.35 | 2.57 | 2.31 | 0.13 |
| ***135*** | 2.80 | 3.23 | 2.86 | 2.92 | 2.79 | 0.16 |
| ***136*** | 1.61 | 1.45 | 1.81 | 1.49 | 1.63 | 0.13 |
| ***137*** | 3.33 | 3.43 | 3.36 | 3.50 | 3.38 | 0.06 |
| ***138*** | 3.96 | 3.91 | 3.96 | 4.08 | 3.91 | 0.06 |
| ***139*** | 3.76 | 3.89 | 3.86 | 3.96 | 3.93 | 0.07 |
| ***140*** | 4.50 | 4.29 | 4.51 | 4.38 | 4.57 | 0.10 |
| ***141*** | 1.91 | 1.86 | 1.70 | 2.32 | 1.88 | 0.21 |
| ***142*** | 3.27 | 3.51 | 2.97 | 2.99 | 3.25 | 0.20 |
| ***143*** | 1.82 | 1.83 | 1.98 | 1.93 | 1.89 | 0.06 |
| ***144*** | 1.91 | 1.83 | 1.86 | 1.86 | 1.77 | 0.05 |
| ***145*** | 3.25 | 3.19 | 2.92 | 3.41 | 3.09 | 0.16 |
| ***146*** | 0.34 | 0.35 | 0.39 | 0.15 | 0.35 | 0.08 |
| ***147*** | 0.82 | 0.83 | 0.67 | 0.94 | 0.63 | 0.11 |
| ***148*** | 1.50 | 1.51 | 1.48 | 1.42 | 1.51 | 0.04 |
| ***149*** | 0.34 | 0.34 | 0.37 | 0.44 | 0.29 | 0.05 |
| ***150*** | 2.43 | 2.46 | 2.74 | 2.24 | 2.83 | 0.21 |
| ***151*** | 2.32 | 2.31 | 2.63 | 2.22 | 2.68 | 0.19 |
| ***152*** | 2.44 | 2.57 | 2.65 | 2.38 | 2.47 | 0.10 |
| ***153*** | 1.33 | 1.38 | 1.36 | 1.29 | 1.44 | 0.05 |
| ***154*** | 3.36 | 3.35 | 3.65 | 3.21 | 3.76 | 0.21 |
| ***155*** | 1.04 | 1.04 | 1.38 | 0.97 | 1.12 | 0.14 |
| ***156*** | 2.82 | 2.98 | 3.18 | 2.63 | 3.23 | 0.22 |
| ***157*** | 3.28 | 3.27 | 3.44 | 3.32 | 3.40 | 0.07 |
| ***158*** | 1.96 | 1.99 | 1.82 | 1.93 | 2.42 | 0.20 |
| ***159*** | 1.27 | 1.32 | 1.39 | 1.13 | 1.74 | 0.20 |
| ***160*** | 2.18 | 2.07 | 2.11 | 2.25 | 2.06 | 0.07 |
| ***161*** | 2.33 | 2.39 | 2.15 | 2.29 | 2.43 | 0.10 |
| ***162*** | 2.77 | 2.91 | 2.70 | 2.71 | 2.94 | 0.10 |
| ***163*** | 2.23 | 2.04 | 2.24 | 2.35 | 1.86 | 0.17 |
| ***164*** | 3.10 | 3.16 | 3.33 | 2.97 | 3.29 | 0.13 |
| ***165*** | 2.13 | 2.25 | 1.95 | 2.18 | 2.00 | 0.11 |
| ***166*** | 1.98 | 1.79 | 2.12 | 2.07 | 1.60 | 0.19 |
| ***167*** | 3.11 | 3.10 | 3.07 | 3.10 | 3.26 | 0.07 |
| ***168*** | 1.55 | 1.62 | 1.41 | 1.60 | 1.36 | 0.10 |
| ***169*** | 2.77 | 2.67 | 2.50 | 2.86 | 2.38 | 0.18 |
| ***170*** | 2.90 | 2.98 | 2.70 | 2.99 | 2.84 | 0.11 |
| ***171*** | 2.40 | 2.37 | 2.20 | 2.46 | 2.43 | 0.09 |
| ***172*** | 2.55 | 2.52 | 2.30 | 2.68 | 2.46 | 0.12 |
| ***173*** | 1.73 | 1.81 | 1.46 | 1.89 | 1.17 | 0.27 |
| ***174*** | 3.78 | 3.92 | 3.56 | 3.71 | 3.71 | 0.11 |
| ***175*** | 4.05 | 3.92 | 4.12 | 3.82 | 3.78 | 0.13 |
